# Naturally Occurring Mutations of SARS-CoV-2 Main Protease Confer Drug Resistance to Nirmatrelvir

**DOI:** 10.1021/acscentsci.3c00538

**Published:** 2023-07-24

**Authors:** Yanmei Hu, Eric M. Lewandowski, Haozhou Tan, Xiaoming Zhang, Ryan T. Morgan, Xiujun Zhang, Lian M. C. Jacobs, Shane G. Butler, Maura V. Gongora, John Choy, Xufang Deng, Yu Chen, Jun Wang

**Affiliations:** † Department of Medicinal Chemistry, Ernest Mario School of Pharmacy, Rutgers, the State University of New Jersey, New Brunswick, New Jersey 08854, United States; ‡ Department of Molecular Medicine, Morsani College of Medicine, University of South Florida, Tampa, Florida 33612, United States; § Department Physiological Sciences, College of Veterinary Medicine, Oklahoma State University, Stillwater, Oklahoma 74078, United States; ∥ Oklahoma Center for Respiratory and Infectious Diseases, Oklahoma State University, Stillwater, Oklahoma 74078, United States; ⊥ Department Biology, School of Arts and Sciences, the Catholic University of America, Washington, DC 20064, United States

## Abstract

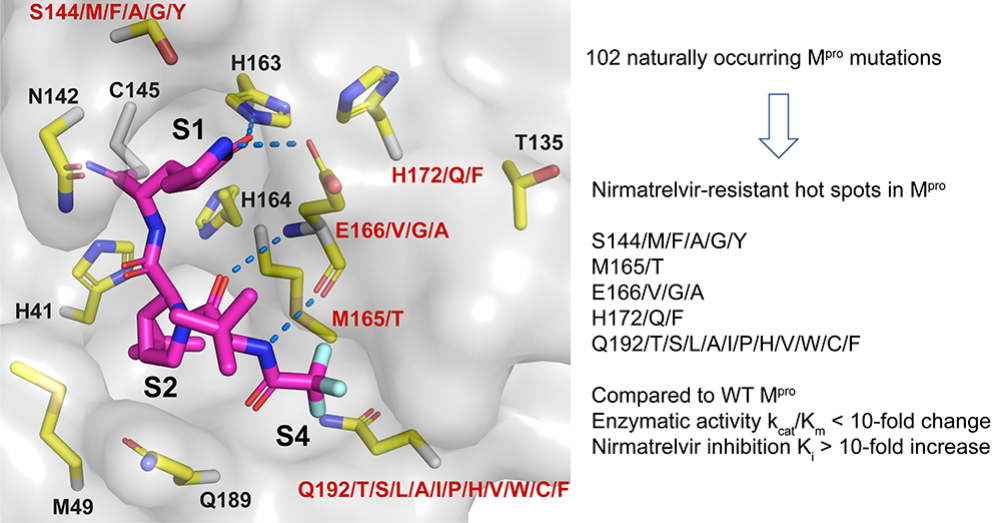

The SARS-CoV-2 main protease (M^pro^) is the drug target of Pfizer’s oral drug nirmatrelvir. The emergence of SARS-CoV-2 variants with mutations in M^pro^ raised the alarm of potential drug resistance. To identify potential clinically relevant drug-resistant mutants, we systematically characterized 102 naturally occurring M^pro^ mutants located at 12 residues at the nirmatrelvir-binding site, among which 22 mutations in 5 residues, including S144M/F/A/G/Y, M165T, E166 V/G/A, H172Q/F, and Q192T/S/L/A/I/P/H/V/W/C/F, showed comparable enzymatic activity to the wild-type (*k*_cat_/*K*_m_ < 10-fold change) while being resistant to nirmatrelvir (*K*_i_ > 10-fold increase). X-ray crystal structures were determined for six representative mutants with and/or without GC-376/nirmatrelvir. Using recombinant SARS-CoV-2 viruses generated from reverse genetics, we confirmed the drug resistance in the antiviral assay and showed that M^pro^ mutants with reduced enzymatic activity had attenuated viral replication. Overall, our study identified several drug-resistant hotspots in M^pro^ that warrant close monitoring for possible clinical evidence of nirmatrelvir resistance, some of which have already emerged in independent viral passage assays conducted by others.

## Abstract 2

Systematic analysis of SARS-CoV-2 main protease sequences in the GISAID database revealed naturally occurring mutations that are resistant to nirmatrelvir without compromised fitness of replication.

## Introduction

The ongoing COVID-19 pandemic highlights the urgent need for oral bioavailable antiviral drugs. Paxlovid combines the viral main protease (M^pro^ or 3CL^pro^) inhibitor nirmatrelvir and its metabolic enhancer ritonavir.^1,2^ Paxlovid was approved by the FDA in 2021 for the treatment of mild-to-moderate COVID-19 in adults and children 12 years old or older with a positive test and who are at high risk of progression to severe COVID-19. M^pro^ is a cysteine protease that mediates the cleavage of viral polyproteins during viral replication and is a high-profile antiviral drug target.^3,4^ In addition to nirmatrelvir, other M^pro^ inhibitors that advanced to the clinical stage include PF-07304814 (phosphate form of PF-00835231), S-217622, PBI-0451, EDP-235, and 13b.^5^ The recent emergence of variants of concern, particularly the Omicron variant, raises the concern of possible altered susceptibility to vaccines and antiviral drugs. Predicting drug resistance before it becomes dominant in clinics is vital in facilitating antiviral drug development to combat the pandemic.

Several recent studies reported the discovery of nirmatrelvir-resistant M^pro^ mutants using the viral passage experiments and identified mutations in residues S144, E166, and A173 that directly affect nirmatrelvir inhibition, as well as other compensatory mutations at residues L50 and T21 that improve viral fitness but have minimal impact on inhibitor binding.^6−9^ Since SARS-CoV-2 continues to evolve with or without selection pressure,^10^ we took an independent and systematic approach to identify naturally occurring drug-resistant M^pro^ mutations by exploiting the SARS-CoV-2 polymorphisms deposited in the Global Initiative on Sharing Avian Influenza Data (GISAID) database. As the sequences in GISAID might contain mutations from noninfectious viruses, our goal is to identify M^pro^ drug-resistant mutations with comparable enzymatic activity as the wild-type (WT). It is known that mutations with a significant reduction of enzymatic activity generally lead to attenuated viral replication.^11^ For this, we focus on mutations with similar enzymatic activity as the wild-type (*k*_cat_/*K*_m_ < 10-fold change) while being resistant to nirmatrelvir (*K*_i_ > 10-fold increase). *k*_cat_ is the catalytic constant, *K*_m_ is the Michaelis–Menten constant, and *K*_i_ is the inhibition constant. In total, we systematically characterized the enzymatic activity, thermal stability, and drug sensitivity of 102 purified M^pro^ mutants that are located within 6 Å of the nirmatrelvir-binding site. While the majority of the M^pro^ mutants showed a significant reduction of enzymatic activity (*k*_cat_/*K*_m_ > 10-fold change compared to WT), we discovered 22 mutations in 5 residues that meet our criteria (*k*_cat_/*K*_m_ < 10-fold change and *K*_i_ > 10-fold increase). The X-ray crystal structures of the representative M^pro^ mutants S144A, S144L, M165Y, E166Q, H172Y, and Q192T with and/or without GC-376/nirmatrelvir provide a structural explanation for the drug resistance. In addition, the viral growth kinetics and drug resistance were characterized using recombinant SARS-CoV-2 viruses with selected M^pro^ mutants. Taken together, this study identified several nirmatrelvir-resistant hotspots that warrant close monitoring while highlighting the future risk of mutants with multiple substitutions at these sites that can directly impart drug resistance, combined with other sites that may enhance viral fitness.

## Results and Discussion

### Identification of SARS-CoV-2 M^pro^ Mutants from GISAID Sequence Analysis

**Figure 1:**
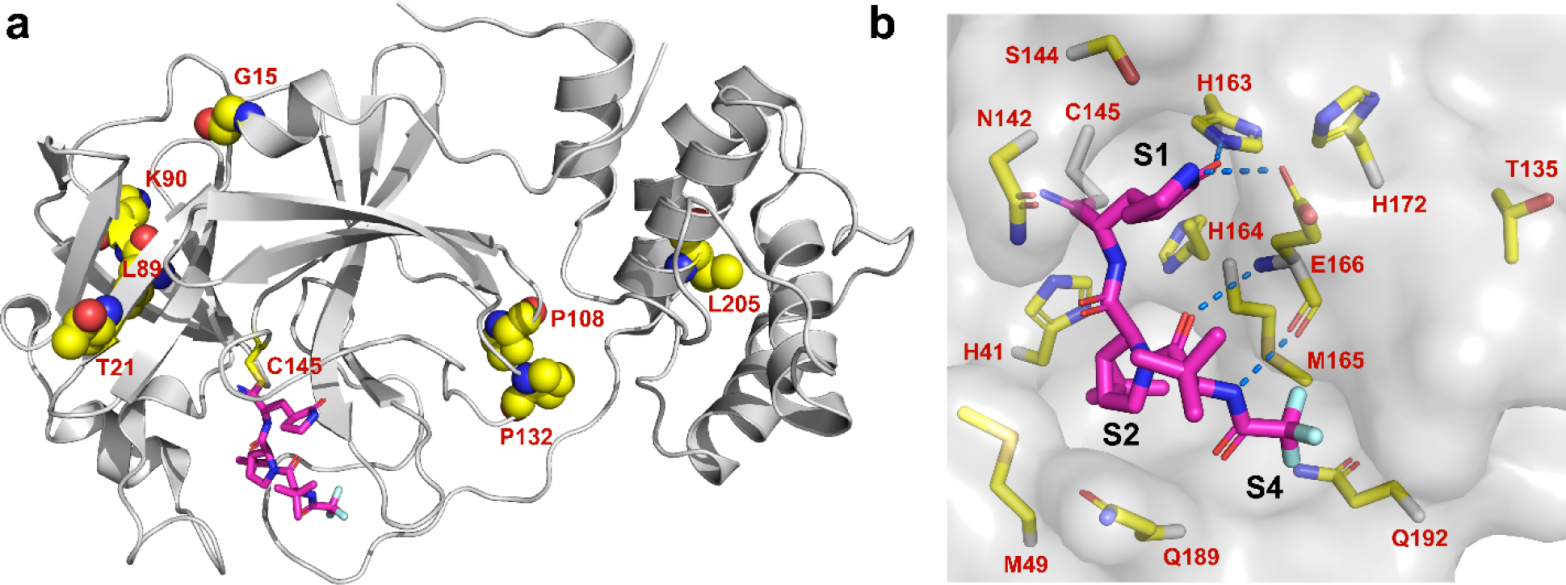
SARS-CoV-2 M^pro^ mutants identified from GISAID sequence analysis. (a) Residues with high mutation rates that were previously examined. None of the mutants showed significant drug resistance. (b) Residues located within 6 Å of the nirmatrelvir-binding site that were examined in this study. Figures were generated using Pymol with the X-ray crystal structure of nirmatrelvir in a complex with SARS-CoV-2 M^pro^ (PDB: 7SI9). Nirmatrelvir is colored magenta.

Recent sequence analysis of SARS-CoV-2 M^pro^ revealed multiple prevalent mutations including G15S, T21I, L89F, K90R, P108S, P132H, and L205V.^12−14^ All these mutants are located outside the nirmatrelvir-binding site (Figure 1a) and were found to have similar catalytic efficacy (*k*_cat_/*K*_m_) as the wild-type (WT) protein.^12,15^ These mutants remained susceptible to nirmatrelvir with no significant IC_50_ or *K*_i_ value shifts (<2-fold).^12^ Nevertheless, drug resistance to nirmatrelvir is anticipated, given the experience from the clinical use of HIV and HCV protease inhibitors.^16,17^ Several studies have been conducted to evolve or predict nirmatrelvir-resistant M^pro^ mutants.^6−9,18−23^ In parallel, we took an independent approach and systematically characterized drug-resistant M^pro^ mutants from naturally occurring SARS-CoV-2 variants using the Nsp5 sequences deposited in the GISAID database.

**Figure 2:**
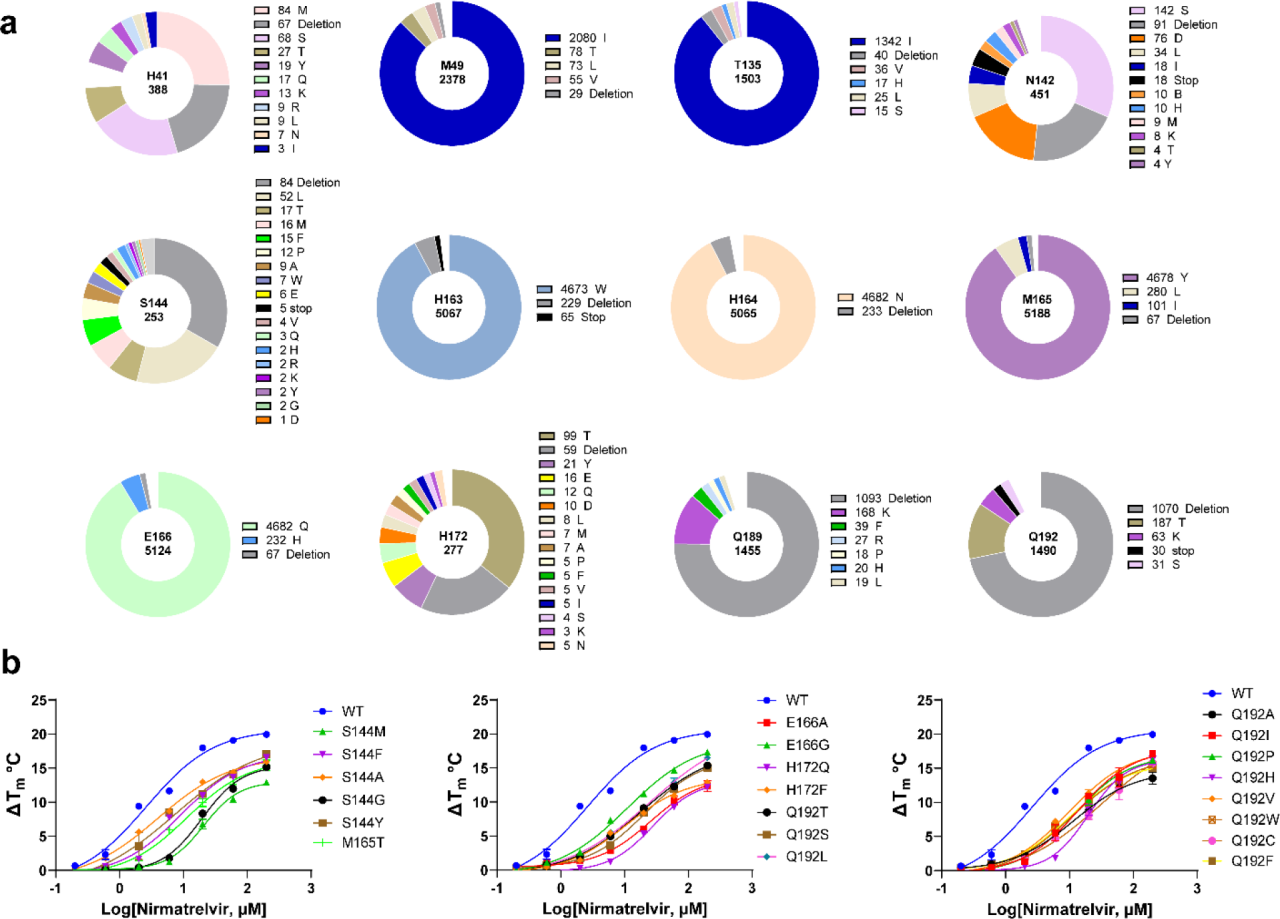
SARS-CoV-2 M^pro^ mutants characterized in this study. (a) Mutations at 12 residues located at the nirmatrelvir-binding site. Sequence data were obtained from CoVsurver of the GISAID, developed by A*STAR Bioinformatics Institute (BII), Singapore. A total of 5 420 461 mutations of Nsp5 (M^pro^) were obtained from the database as of July 7, 2022. Occurrence of total mutations for each amino acid residue is labeled in the center of each pie chart. The occurrences of specific mutations are labeled on the right of its affiliate pie chart. (b) Characterization of nirmatrelvir resistance against M^pro^ mutants by the thermal shift assay. The results are the average of duplicates.

To identify drug-resistant mutants of M^pro^, we focus on the active site residues that are located within 6 Å of the nirmatrelvir-binding site (PDB: 7SI9).^2^ In total, 12 residues were selected, including H41, M49, T135, N142, S144, H163, H164, M165, E166, H172, Q189, and Q192 (Figure 1b). We expect that mutations at these active site residues will have a direct impact on substrate binding and drug inhibition. To test this hypothesis, we analyzed the mutations of these 12 residues using the SARS-CoV-2 sequences deposited in GISAID,^24^ and the mutation frequency of each active site residue is plotted in Figure 2a.

We chose 102 mutants that cover all high-frequency mutations at these 12 residues based on the sequence analysis. Although the GISAID sequences are from clinical samples, not all mutants are likely from viable or infectious SARS-CoV-2 viruses, and the database might contain sequences from nonreplicating viruses or even sequencing errors (Figure 2a). As such, it is critical to experimentally characterize the M^pro^ mutations and identify the ones with potential clinical relevance. For this, 102 tag-free recombinant SARS-CoV-2 M^pro^ mutants with native N- and C-termini were expressed in *Escherichia coli* and purified (Figure S1). The majority of the mutants were folded correctly as shown by the thermal shift assay (Figure S2) and the enzymatic assay (Table S1). The enzymatic activity (*k*_cat_/*K*_m_) of the mutant proteins was determined using the FRET assay.^25,26^ Three inhibitors—GC-376, PF-00835231, and nirmatrelvir—were examined for drug resistance (Figure S3). GC-376 is a veterinary drug candidate for treating cats’ feline infectious peritonitis virus (FIPV) infection.^27^ The phosphate prodrug of PF-00835231 was a clinical candidate developed by Pfizer as an intravenous drug for treating COVID patients in hospitals.^28^ The drug sensitivity was characterized using a FRET-based enzymatic assay (Table S1) and the thermal shift binding assay (Figure 1b).

To profile the drug resistance, we first tested the M^pro^ mutants against nirmatrelvir in the FRET assay and determined the half-maximal inhibitory concentration (IC_50_) and inhibition constant (*K*_i_). The *K*_i_ value is protein concentration independent and was used for selection of M^pro^ mutants with a significant drug resistance (*K*_i_ > 10-fold increase). For mutants showing resistance against nirmatrelvir, the drug sensitivity was further tested against PF-00835231 and GC-376 for cross-resistance. The comprehensive data set is shown in Table S1.

### H41 and H163 Are Critical for the Enzymatic Activity

Among the 102 mutants, H41M, H41T, H41Y, and H163W were enzymatically inactive (Table S1, yellow), which validates our hypothesis that not all mutants listed in the GISAID database are from infectious SARS-CoV-2 viruses. H41 forms the catalytic dyad with C145, and all three mutants—H41M (84 occurrences), H41T (27 occurrences), and H41Y (19 occurrences)—were detrimental to the enzymatic activity (Table S1, yellow). The X-ray crystal structure showed that the side chain imidazole of H163 forms a hydrogen bond with the carbonyl from the nirmatrelvir P1 pyrrolidone (Figure 1b) (PDB: 7SI9)^2^ or similar functional groups from other inhibitors, suggesting its essential contribution to drug binding. Although H163W is a high-frequency mutation with 4673 occurrences, this mutant led to an inactive enzyme.

### S144, M165, E166, H172, and Q192 Are Hotspots for Drug Resistance

It is generally assumed that mutations with impaired enzymatic activity will lead to attenuation of viral replication.^11^ We therefore focused on M^pro^ mutants with *k*_cat_/*K*_m_ values within 10-fold variation compared to WT. For drug sensitivity, we define a *K*_i_ increase by more than 10-fold as a significant resistance. In total, 22 M^pro^ mutants met both criteria including S144M, S144F, S144A, S144G, S144Y, M165T, E166V, E166G, E166A, H172Q, H172F, Q192T, Q192S, Q192L, Q192A, Q192I, Q192P, Q192H, Q192V, Q192W, Q192C, and Q192F (Table S1, red).

S144 is located at the S1 pocket and is part of the oxyanion hole consisting of two additional residues G143 and C145 (Figure 1b). Among the top 15 high-frequency mutations at S144, five mutants—S144M (8.0-fold lower *k*_cat_/*K*_m_), S144F (5.8-fold), S144A (1.8-fold), S144G (2.6-fold), and S144Y (7.8-fold)—had comparable enzymatic activity to the WT. Significantly, all five mutants showed drug resistance against nirmatrelvir with *K*_i_ values increased between 19.2- and 38.0-fold. Pfizer’s report for healthcare providers similarly disclosed S144A as a nirmatrelvir-resistant mutant with a *K*_i_ increase of 91.9-fold^29^ compared to 20.5-fold from our study. S144A was also identified from the SARS-CoV-2 viral passage experiment with nirmatrelvir,^8^ corroborating the significance of our approach in identifying clinically relevant nirmatrelvir-resistant SARS-CoV-2 mutants. Four mutants—S144L (183.3-fold lower in *k*_cat_/*K*_m_), S144P (523.8-fold), S144R (478.3-fold), and S144K (534.0-fold)—had significantly reduced enzymatic activity compared to WT and increased resistance to nirmatrelvir. Similarly, the remaining seven mutants—S144T, S144W, S144E, S144V, S144Q, S144H, and S144D—had compromised enzymatic activity with *k*_cat_/*K*_m_ values decreased between 20.0- and 85.9-fold compared to WT. A significant drug resistance against nirmatrelvir was also observed for these seven mutants.

M165 is located at the S2 pocket and forms a hydrophobic interaction with the P2 dimethylcyclopropylproline (Figure 1b). The most frequent mutant, M165Y, had a significantly reduced enzymatic activity (41.7-fold decrease in *k*_cat_/*K*_m_), while M165L, M165I, M165V, M165T, M165A, and M165C had similar enzymatic activity as the WT. No drug resistance was observed for M165L, M165I, M165V, M165A, and M165C. However, a significant drug resistance against nirmatrelvir was observed for M165T (29.9-fold increase in *K*_i_). The remaining mutants M165W/K/R/G/F/H/P/D had a significantly reduced enzymatic activity (>14-fold decrease in *k*_cat_/*K*_m_).

E166 is located at the S1 pocket and is a critical residue for drug binding as it forms three hydrogen bonds with nirmatrelvir (Figure 1b). E166Q is a high-frequency mutation with 4682 occurrences. It has a similar enzymatic activity (*k*_cat_/*K*_m_) as the WT, and no significant drug resistance against nirmatrelvir was observed (4.5-fold increase in *K*_i_). E166H/K/L/Y/I/V mutants all had a significant reduction of enzymatic activity (>17.5-fold decrease in *k*_cat_/*K*_m_) and a high degree of drug resistance. Interestingly, E166G only had a 7.4-fold reduction in the enzymatic activity *k*_cat_/*K*_m_ value but a 16.4-fold increase in the *K*_i_ value against nirmatrelvir. Similarly, E166A had comparable enzymatic activity as the WT (7.5-fold decrease in *k*_cat_/*K*_m_) but a significant drug resistance against nirmatrelvir (47.5-fold increase in *K*_i_). E166V showed nearly complete resistance (*K*_i_ > 10 μM). In parallel to our study, three groups similarly identified E166A and E166V as nirmatrelvir-resistant M^pro^ mutants using serial viral passage experiments in cell culture with infectious SARS-CoV-2 virus.^6−8^ Jochmans et al. discovered a triple mutant, L50F/E166A/L167F, with a 72-fold IC_50_ increase in the enzymatic assay and a 51-fold EC_50_ increase in the antiviral assay against nirmatrelvir.^7^ In another study, Zhou et al. showed that the L50F/E166V double mutant led to an 80-fold resistance in the antiviral assay against nirmatrelvir.^6^ Iketani et al. similarly identified E166V as a nirmatrelvir-resistant mutant from the SARS-CoV-2 viral passage experiment.^8^ However, the drug resistance of E166V and E166A mutations was not fully characterized by enzymatic assay in these studies. Our result confirmed that E166A and E166V indeed confer drug resistance. Both E166A and E166V are naturally occurring mutations with five and eight occurrences. Taken together, E166 appears to be a hotspot for drug resistance, which has been confirmed by several independent studies.^6−8^

H172 locates at the S1 pocket but does not directly interact with nirmatrelvir (Figure 1b). Among the 17 H172 mutants examined, H172Q (3.2-fold lower *k*_cat_/*K*_m_) and H172F (9.9-fold) had comparable enzymatic activity as the WT. Both mutants showed significant drug resistance against nirmatrelvir (>42-fold increase in *K*_i_). The H172Y (13.9-fold lower *k*_cat_/*K*_m_) and H172A (11.3-fold lower *k*_cat_/*K*_m_) mutants had reduced enzymatic activity, while being resistant to nirmatrelvir (>113.7-fold increase in *K*_i_). Pfizer similarly disclosed H172Y as a nirmatrelvir-resistant mutant (233-fold increase in *K*_i_).^29^ The remaining mutants H172T/E/D/L/M/I/V/S/N/K/R/G/C had a significantly reduced enzymatic activity (>21.0-fold lower *k*_cat_/*K*_m_).

Q192 locates at the S4 pocket and forms a hydrophobic interaction with the trifluoromethyl substitution from nirmatrelvir (Figure 1b). Q192T (9.2-fold lower *k*_cat_/*K*_m_), Q192S (8.9-fold), Q192L(4.3-fold), Q192A (6.2-fold), Q192I (5.6-fold), Q192P (7.6-fold), Q192H (8.2-fold), Q192V (9.0-fold), Q192W (8.0-fold), Q192C (7.0-fold), and Q192F (3.5-fold) had comparable enzymatic activity as the WT, and all showed resistance against nirmatrelvir (>22.2-fold increase in *K*_i_). Cross-resistance was also observed with PF-00835231 (>25.5-fold) and GC-376 (>7.7-fold). Results from Sasi et al. similarly confirmed the drug resistance of Q192T.^20^

Differential scanning fluorimetry (DSF) assay is widely used to determine the direct binding of a protein and a ligand. Binding of a ligand typically stabilizes the target protein, resulting in an increased melting temperature (*T*_m_). The larger degree of *T*_m_ shift, the tighter the binding of the ligand. The drug resistance of these 22 M^pro^ mutants against nirmatrelvir was further confirmed in the thermal shift drug titration assay. All mutants displayed a lower degree of protein stabilization than the WT M^pro^ with increasing concentrations of nirmatrelvir (Figure 2b) as decreased Δ*T*_m_ was observed, indicating the binding of nirmatrelvir to the mutants is weakened compared to the WT M^pro^. The Δ*T*_m_ values of WT and mutant M^pro^ proteins at different concentrations of nirmatrelvir are listed in Table S2.

### M49, T135, N142, H164, M165, and Q189 Can Tolerate Multiple Mutations without Significantly Affecting the Enzymatic Activity and Drug Inhibition

The most frequent mutants at residue M49—M49I, M49T, M49L, and M49V—remained sensitive to nirmatrelvir (<3-fold change in IC_50_). Interestingly, the enzymatic activity (*k*_cat_/*K*_m_) of the M49I and M49L mutants showed a 1.69 and 1.74-fold increase compared to WT.

T135I is a high-frequency mutation with 1342 occurrences. The T135I mutant had a similar *k*_cat_/*K*_m_ value as the WT and remained sensitive to all three inhibitors (<2.9-fold change in *K*_i_).

The top nine high-frequency mutants at residue N142 all had similar enzymatic activity as the WT (<4.1-fold change in *k*_cat_/*K*_m_) and remained sensitive to nirmatrelvir (<3.5-fold change in IC_50_).

H164N is a high-frequency mutation with 4682 occurrences and remained sensitive to all three inhibitors (<4.1-fold change in *K*_i_). The catalytic activity of the H164N mutant (4.2-fold lower in *k*_cat_/*K*_m_) is comparable to that of the WT.

All eight Q189 mutants retained similar enzymatic activity as the WT with the change in *k*_cat_/*K*_m_ values between 1.9- and 9.2-fold. No significant drug resistance was observed for nirmatrelvir (<3.1-fold change in IC_50_). Interestingly, the enzymatic activity of Q189E increased by nearly twofold compared to WT.

Collectively, the results suggest that M49, T135, N142, H164, and Q189 might be able to accommodate multiple mutations without a significant compromise in enzymatic activity and drug sensitivity. Nevertheless, we cannot rule out the possibility that other mutants at these residues that are not covered in this study might cause drug resistance.

### M^pro^ Deletion Mutations

It is noticed that deletions frequently occur on the residues at the nirmatrelvir-binding site (Figure 2a). To validate the effects of deletion on the enzymatic activity and nirmatrelvir inhibition, we expressed, purified, and characterized 10 M^pro^ deletion mutations: M49del, T135del, N142del, S144del, H164del, M165del, E166del, H172del, Q189del, and Q192del. As expected, all the deletion mutants except M49del had significantly reduced enzymatic activity (*k*_cat_/*K*_m_ ≤ 69 S^–1^ M^–1^) (Table S1). Interestingly, M49del remains enzymatically active (*k*_cat_/*K*_m_ = 5653 vs 11 000 S^–1^ M^–1^ for WT) and is also sensitive to nirmatrelvir binding (*K*_i_ = 8.02 ± 0.82 vs 1.88 nM for WT).

### Recombinant SARS-CoV-2 Viruses with M^pro^ S144 M or H172Y/Q Mutants Had Increased Resistance but Attenuated Replication in Cell Culture

**Figure 3:**
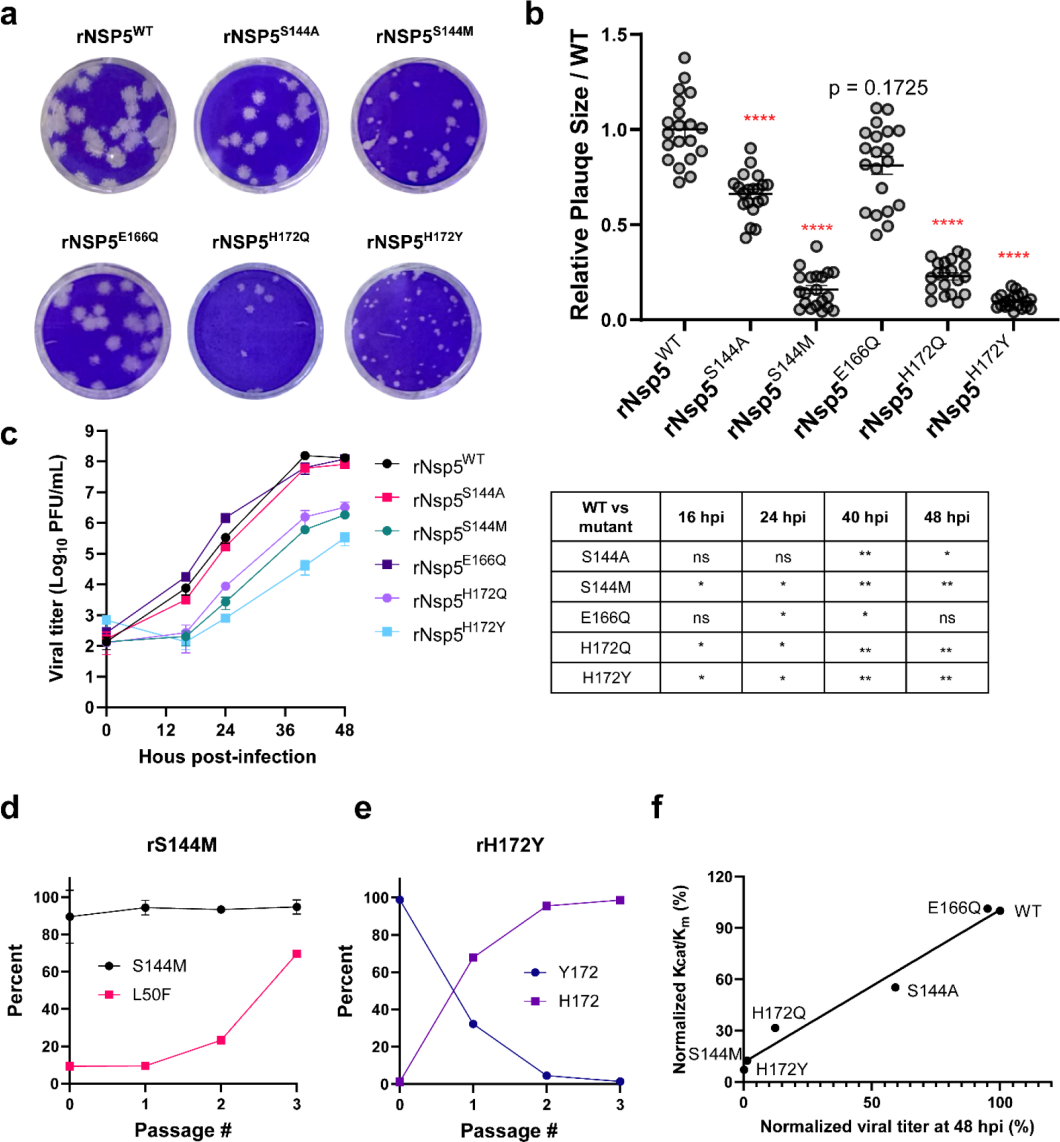
Characterization of the recombinant SARS-CoV-2 Nsp5 mutants. (a) Plaque formation by recombinant SARS-CoV-2 wild-type (rNsp5^WT^) and Nsp5 mutant viruses. (b) Sizes of 20 plaques from the same batch plaque assay were measured for each virus using ImageJ. Relative plaque sizes over WT are presented as mean ± SEM. Statistical significance of the size differences of each virus vs rNsp5^WT^ virus was determined using the unpaired Kolmogorov–Smirnov test in Prism 9. Statistical significance is demarcated with red asterisks. ****P ≤ 0.0001; ns: not significant. (c) Growth kinetics of rNsp5^WT^ and Nsp5 mutant viruses in Vero-E6 expressing hTMPRSS2 and hACE2 (Vero-TA). Cells were infected with the indicated virus at an MOI of 0.001. The culture supernatants were collected at indicated times and titrated in Vero-TA cells using plaque assay. The graph represents the titers obtained from three biological replicates (mean ± SD). Statistical significance of titer differences of each Nsp5 mutant virus compared to Nsp5 WT virus was calculated by repeated measures two-way ANOVA: *P ≤ 0.05; **P ≤ 0.01. Data that were not statistically significant are labeled ns. (d and e) Passage experiments of rNsp5^S144M^ and rNsp5^H172Y^ in Vero-TA cells revealed the proportion of each genotype in each passage. The percentage of each genotype was determined by analyzing the chromatographic values of each nucleotide in the sequencing trace files. (f) Correlation plot between the normalized *k*_cat_/*K*_m_ and the normalized viral titer at 48 hpi for the six recombinant SARS-CoV-2 mutants. All experiments were independently performed at least twice. Data are shown as mean ± SD.

To investigate the effect of M^pro^ mutants on viral replication and the sensitivity to nirmatrelvir, we characterized four representative mutants S144A/M and H172Y/Q. We also included E166Q as a control because it has similar *k*_cat_/*K*_m_ and IC_50_ as the WT M^pro^. To this end, we successfully generated all five recombinant SARS-CoV-2 viruses harboring the S144A/M, E166Q, and H172Y/Q mutations, designated rNsp5^S144A^, rNsp5^S144M^, rNsp5^E166Q^, rNsp5^H172Q^, and rNsp5^H172Y^, respectively, using a SARS-CoV-2 reverse genetic system.^30^ An isogenic wild-type recombinant SARS-CoV-2 WA1 strain (rNsp5^WT^) was also generated and served as a wild-type control. The Nsp5 coding sequences of these recombinant viruses were sequenced, and the corresponding mutants were confirmed. We first performed plaque assay and growth kinetics analysis to evaluate viral replication. As expected, rNsp5^E166Q^ formed similar sizes of plaques (Figure 3a and b) and had comparable growth kinetics (Figure 3c) as the rNsp5^WT^, which was expected as it has similar *k*_cat_/*K*_m_ as the WT M^pro^ (Table S1). As shown in Figure 3a and b, Nsp5^S144A^ mutant formed statistically smaller plaques as the rNsp5^WT^, whereas the plaques of rNsp5^S144M^, rNsp5^H172Q^, and rNsp5^H172Y^ mutants were drastically smaller than those of rNsp5^WT^. Growth kinetics analysis revealed that the rNsp5^E166Q^ had similar growth kinetics as the rNsp5^WT^, while rNsp5^S144A^ had slightly lower titers at the plateau growth phase (Figure 3c). In stark contrast, the rNsp5^S144M^, rNsp5^H172Q^, and rNsp5^H172Y^ mutants exhibited a significant replication defect and had 2–4-log lower titers at the exponential growth phase compared to rNsp5^WT^. Passage experiments revealed that after three passages rNsp5^S144M^ and rNsp5^H172Y^ formed similar-size plaques as rNsp5WT did in Vero-TA cells (Figure S4), suggesting these mutant viruses regain the replicative capacity. Sequencing analyses showed that rNsp5^S144M^ gradually accumulated a secondary mutation L50F, while rNsp5^H172Y^ rapidly reversed Y172 back to H172 within three passages (Figure 3d and e). These data together demonstrate that the E166Q and S144A mutations did not significantly impair viral growth, whereas the S144M, H172Q, and H172Y mutations severely weakened viral replication, consistent with the *in vitro* enzymatic analysis results (Table S1). To further delineate the relationship between the catalytic activity *k*_cat_/*K*_m_ and the replication efficiency, we generated a correlation plot between the normalized viral titers at 48 hpi and the normalized *k*_cat_/*K*_m_. The correlation plot showed that mutants with reduced enzymatic activity (*k*_cat_/*K*_m_) had slower replication kinetics (Figure 3f).

**Figure 4:**
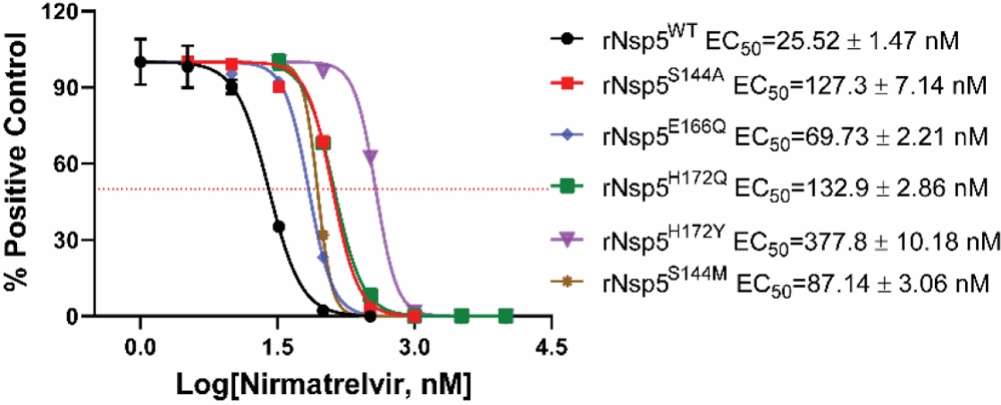
Drug resistance of recombinant Nsp5 mutant viruses. Antiviral CPE assay of rNsp5^WT^ and Nsp5 mutant viruses in Vero-TA cells.

Next, we assessed the sensitivity to nirmatrelvir and performed antiviral experiments using the plaque assay in Vero-TA cells. As shown in Figure 4, the EC_50_ value of nirmatrelvir against rNsp5^S144A^ was 127.3 nM, 5.0-fold higher than the EC_50_ (25.5 nM) of rNsp5^WT^. The antiviral EC_50_ value of nirmatrelvir against rNsp5^E166Q^ was 69.7 nM, a 2.7-fold increase compared to rNsp5^WT^, which is consistent with the enzymatic assay results (Table S1). rNsp5^S144M^ and rNsp5^H172Q^ also showed increased resistance against nirmatrelvir with the EC_50_ value increased by 3.4- and 5.2-fold, respectively.

### Structural Basis for Resistance Mutations

**Figure 5:**
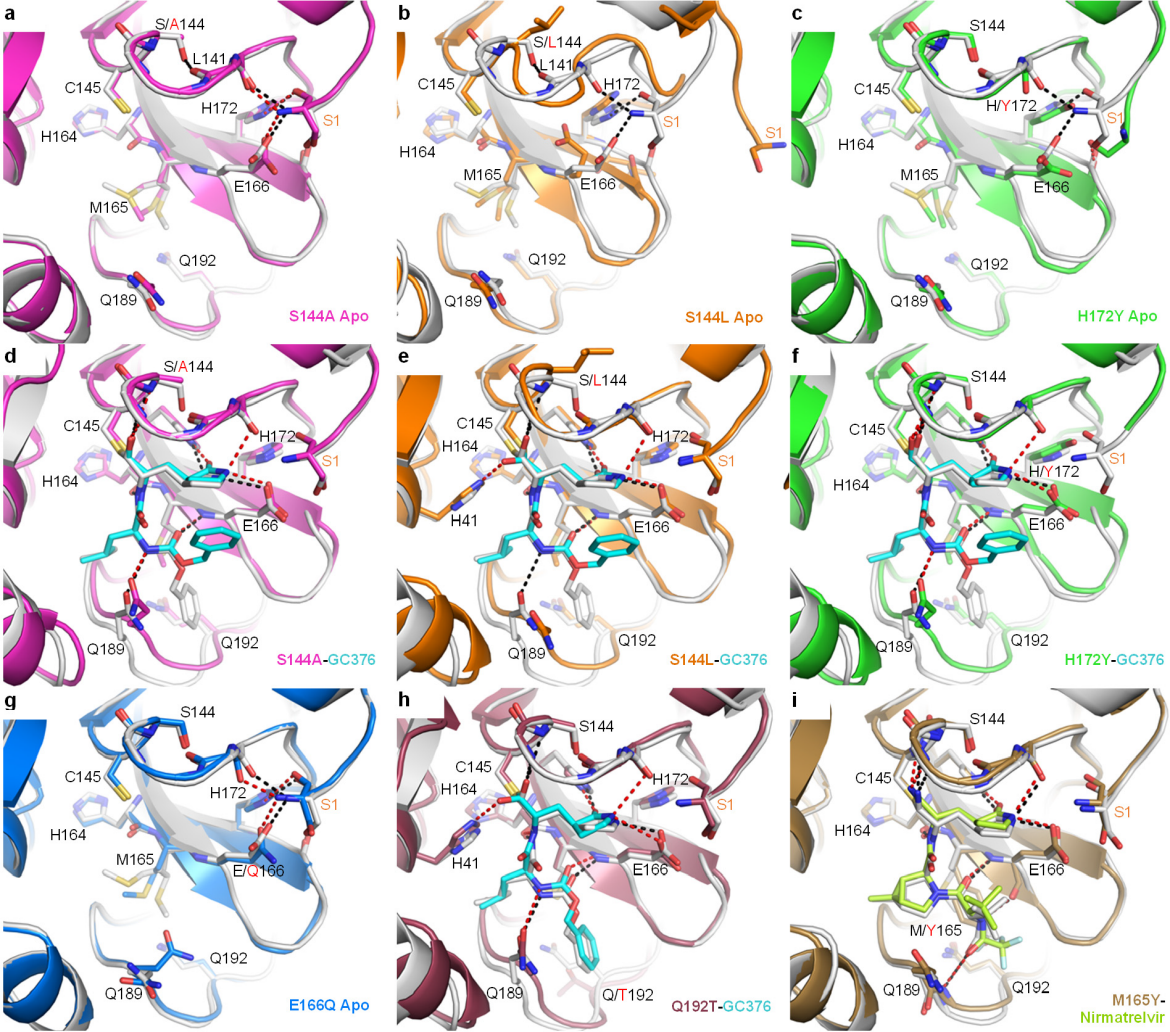
X-ray crystal structures of SARS-CoV-2 M^pro^ mutants. Each mutant structure is aligned with the corresponding WT structure shown in white (apo, PDB 7JP1; GC-376 complex, PDB 6WTT; nirmatrelvir complex, PDB 7RFW). For the mutant structures, GC-376 and nirmatrelvir are shown in cyan and neon green, respectively. WT HBs are shown as black dashes for selected residues at the mutation sites or between the protein and inhibitor. Mutant HBs are shown as red dashes. Mutations are indicated with red text. The S1 residue from the N-terminus of the adjacent protomer is labeled in orange. The side chain of L141 is not shown. (a) Apo M^pro^ S144A (magenta, PDB 8D4L). (b) Apo M^pro^ S144L (orange, PDB 8DFE). The view for panel B is shifted slightly to show the movement of the adjacent N-terminus. (c) Apo Mpro H172Y (green, PDB 8D4J). (d) M^pro^ S144A GC-376 complex (magenta, PDB 8D4M). (e) M^pro^ S144L GC-376 complex (orange, PDB 8DD9). (f) M^pro^ H172Y GC-376 complex (green, PDB 8D4K). S1 residue is disordered and not modeled. (g) Apo M^pro^ E166Q (blue, PDB 8D4N). (h) M^pro^ Q192T GC-376 complex (mauve, PDB 8DGB). (i) M^pro^ M165Y nirmatrelvir complex (brown, PDB 8DCZ).

We have determined the X-ray crystal structures of several key representative mutants, including both unbound and GC-376 complex structures for S144A, S144L, H172Y, the GC-376 complex of Q192T, and the nirmatrelvir complex of M165Y, at 1.70–2.87 Å resolutions (Figure 5 and Table S3). The structures of H164N (apo and GC-376 bound) and E166Q (apo) were also determined for comparison (Figure S5). GC-376 and nirmatrelvir place the same pyrrolidone group in the S1 pocket. It should be noted that the terminal benzene group of GC-376 exhibited two different binding modes in previously published WT structures.^25,26^ The conformational difference in this substitution between mutants (except Q192T) and the WT likely originated from its flexibility, rather than the specific mutations.

All the WT residues at the four mutation sites (S144, M165, H172, and Q192) are involved in intramolecular interactions and are at least partially buried. However, except for S144L, the changes caused by the five mutations are mostly local and small. All mutant complex structures including S144L showed the inhibitor and the protein assumed conformations very similar to those of the WT.

The decrease in the mutants’ catalytic activity and inhibition by GC-376/nirmatrelvir appears to stem from two causes, a large enthalpic effect through direct disruption of ligand-binding interactions and an entropic effect through increasing conformational instability of the active site. S144L, M165Y, H172Y, and Q192T represented some of the biggest changes in both the residue size and the enzyme activity, with a decrease of ∼127×, 39×, 13×, and 10×, respectively, in *k*_cat_ values. Those mutations resulted in notable changes in ligand interactions in the structures. In the unbound state (Figure 5b) and, to a lesser extent, the complex structure (Figure 5e), the S144L mutation led to a drastically different conformation in the 140–146 loop, as well as neighboring regions to avoid steric clashes caused by the bulky leucine side chain. Importantly, this loop constitutes the oxyanion hole stabilizing the transition state of the enzymatic reaction formed by the main chain amide groups of G143, S144, and C145. Interestingly, in the GC-376 complex structure of S144L (Figure 5e) as well as Q192T (Figure 5h), the thioacetal hydroxide is placed outside the oxyanion hole and hydrogen bonds with H41, unlike most previously determined structures.^25^ This suggests that the interactions between the oxyanion hole and the inhibitor thioacetal hydroxide, as well as, by extension, the substrate transition state, are likely weakened in the S144L and Q192T mutants, resulting in an alternate conformation in the inhibitor crystal structure. Similarly, the M165Y mutation also seemed to lead to diminished interactions between the oxyanion hole and the ligand, indicated by the lengthened HB between the imine nitrogen and G143 amide NH (from 3.0 Å in the WT to 3.6 Å in the M165Y mutant) (Figure 5i). These distortions are likely caused by the bulky Y165 residue through a series of ripple effects relayed by both the protein and inhibitor in a tightly packed active site. Meanwhile, the H172Y mutation altered the conformation of the N-terminus of the adjacent protomer in the biological dimer, which is near the active site. In addition to these effects on the reaction center, the M165Y and Q192T mutations also directly impacted ligand interactions in the S4 site. The M165Y mutation pushes the terminal trifluoromethyl group of nirmatrelvir out of its original position in the WT complex, disrupting its interaction with L167 and the T190 backbone oxygen (Figure 5l). The Q192T mutation increased the plasticity of the surrounding residues (Figure 5h), allowing them to better accommodate the terminal benzene ring of GC-376, which assumed a different conformation compared with the other mutants.

In contrast to the above four mutations, the remaining resistant mutation, S144A, represented the smallest changes in the side chain size and led to nearly no alteration of the unbound structure compared with the WT, similar to the structure of the E166Q mutant that remained sensitive to nirmatrelvir inhibition (Figure 5a, d, and g). However, the S144 mutant located in the active site abolishes the intramolecular interactions involving the S144 side chain in the WT and subsequently increases the conformational instability of the protein active site. Even though the enthalpic interactions between the substrate/inhibitor and the protein may be similar to the WT in the lowest energy conformation, the entropic cost will be higher for the mutants, thus making the free energy of ligand binding less favorable. As the inhibitor relies on better shape complementarity with a smaller portion of the active site and contains more rigid features than the substrate, we hypothesize that this entropic cost may impact inhibitor binding more than the larger and more flexible substrate. For example, compared with the glutamine side chain at the P1 position, the pyrrolidone ring of nirmatrelvir and GC-376 forms additional interactions with the peptide bond between L141 and N142 through the two extra carbon atoms. The S144A mutation eliminates the HB with the carbonyl group of this peptide bond, likely increasing its flexibility and increasing the energetic cost of inhibitor binding. Such entropic effects are not limited to the S144A mutation causing minimal structural changes but also apply to those aforementioned mutations such as S144L and H165Y that can both directly influence the protein–ligand contacts and increase the active site flexibility.

The cocrystal structures of M^pro^ mutants with GC-376/nirmatrelvir are insightful in guiding the design of the next generation of M^pro^ inhibitors with enhanced genetic barriers to drug resistance. For example, to avoid the drug resistance caused by the Q192 mutant, which is located at the S4 binding pocket (Figure 1b, 5), one can design inhibitors that do not bind to the S4 pocket. Such examples include Calpain inhibitors II and XII.^26^ S144, H164, E166, H172, and N142 mutants impact the size and hydrophobicity of the S1 binding pocket (Figures 1b and 5). As such, various P1 substitutions other than the pyrrolidone such as pyridine or pyrimidine need to be explored.^31^ Q189, M49, H41, and M165 are key residues forming the S2-binding pocket (Figures 1b and 5), and corresponding P2 substitutions such as spiroproline can be considered for inhibitor design.^32^ Additional consideration for the design of M^pro^ inhibitors with a high genetic barrier to drug resistance is to fit inhibitors within the substrate envelope.^19^

## Conclusions

Collectively, our results have several implications. First, all 102 M^pro^ mutants characterized in this study are naturally occurring SARS-CoV-2 M^pro^ polymorphisms that could potentially affect the efficacy of Paxlovid, and continuous prescription of Paxlovid might likely increase the frequency of these pre-existing drug-resistant mutants. Examples of naturally occurring resistant mutants against antivirals include the amantadine-resistant influenza A virus M2-S31N mutant,^33^ the Tamiflu-resistant H275Y mutant,^34^ and the telaprevir-resistant HCV protease mutants,^35^ all of which emerged without drug selection pressure. Second, S144, M165, E166, H172, and Q192 appear to be hotspots for nirmatrelvir resistance and must be closely monitored among circulating viruses. Mutations at these residues are most likely to maintain the enzymatic activity while causing significant drug resistance. As such, the 22 high-profile mutations can serve as markers for monitoring nirmatrelvir resistance in the clinic. Third, although the 22 high-profile M^pro^ mutations in these five hotspot residues have yet become dominant in current circulating viruses, their clinical relevance should not be underestimated. Indeed, three mutations from two of these hotspot residues S144A, E166A, and E166V were also identified as drug-resistant mutations from the SARS-CoV-2 serial viral passage experiments.^6−8^ These results further validated our hypothesis that drug-resistant mutations can emerge with or without drug selection pressure. Fourth, M^pro^ mutants with a significantly reduced enzymatic activity (>10-fold decrease in *k*_cat_/*K*_m_) such as H172Y and S144M impair the fitness of viral replication in cell culture, suggesting the resistance risk due to single mutations may be relatively low. However, as exemplified by the L50F/S144M mutant from this study as well as the L50F/E166V and L50F/E166A/L167F mutants isolated by others,^6−8^ additional complementary mutants can emerge to compensate for the loss of fitness of replication from the single mutant while maintaining or enhancing drug resistance, which significantly raises the resistance risk especially considering the multiple naturally occurring mutations shown by our study to confer resistance. This will be particularly worrisome as Paxlovid is being more widely used. Therefore, the M^pro^ mutants with reduced enzymatic activity from this study should also be monitored. Fifth, our study is by far the most comprehensive study of M^pro^ drug-resistant mutants. Complementary to the limited number of mutations (S144A, E166A, E166V, and L167F) selected from the SARS-CoV-2 passage experiment, our study identified 19 additional high-profile mutants. Such information, including crystal structures, can be used to develop next-generation antivirals.

Nevertheless, it is important to state the limitations of our approach, so the results are not overinterpreted. First, as we mainly focus on naturally occurring M^pro^ mutants with high frequency, we might miss drug-resistant mutants with low frequency or mutations that are not covered by the GISAID database. For this, we will characterize additional mutations in the hotspot residues in the following studies. Second, we did not cover allosteric mutations located outside the drug-binding site, which might similarly cause drug resistance. For this, computational prediction or saturation mutagenesis using yeast systems might have advantages.^20,36,37^ Third, as suggested by the previous serial viral passage experiments,^13,14,21^ additional compensatory mutations outside the nirmatrelvir-binding site such as L50F and T21I might be necessary to restore the reduced fitness of replication of SARS-CoV-2 viruses with the single M^pro^ mutation identified from this study. Fourth, as Omicron is the current dominant circulating strain, M^pro^ drug resistance study should be performed with the P132H background, especially the serial passage experiment.

Overall, our study identified five hotspot residues located at the drug-binding site of nirmatrelvir that warrant close monitoring in the clinical setting. The results also call for the development of the next generation of M^pro^ inhibitors with a high genetic barrier to drug resistance or combination therapy to reduce the incidence of resistance.^38^

## Experimental Section

### FRET Assay, Cell Lines, and SARS-CoV-2 Plasmid Clones

Oligonucleotides were from Integrated DNA Technologies (Coralville, IA). The SARS-CoV-2 M^pro^ FRET substrate Dabcyl-KTSAVLQ/SGFRKME-(Edans) was synthesized as previously described.^25^ This peptide substrate contains a 14 amino acid sequence corresponding to the viral NSP4-NSP5 polypeptide junction and Dabcyl and Edans on its N- and C-terminals, respectively. All other chemicals were purchased from either Sigma-Aldrich (St. Louis, MO) or Fisher Scientific (Pittsburgh, PA). DNA sequencing was performed by Azenta Life Sciences (South Plainfield, NJ). The following reagents were obtained through BEI Resources, NIAID, NIH: Cercopithecus aethiops Kidney Epithelial Cells Expressing Transmembrane Protease, Serine 2 and Human Angiotensin-Converting Enzyme 2 (Vero E6-TMPRSS2-T2A-ACE2, Vero-TA), NR-54970; SARS-Related Coronavirus 2, USA-WA1/2020 Recombinant Infectious Molecular Clone Plasmid Kit, NR-53762. Vero-TA cells were maintained in Dulbecco’s modified Eagle medium (DMEM) (Corning, 10013CM) containing 10% heat-inactivated fetal bovine serum (FBS), 1% Pen/Strep, 1× nonessential amino acid, and 10 μg/mL puromycin (InVivogen, ant-pr-1) to maintain the expression of TMPRSS2 and ACE2. The SARS-CoV-2 infectious plasmid clones were propagated in bacterial cells TOP10 strain (ThermoFisher, C404010) and sequenced.

### SARS-CoV-2 M^pro^ Mutagenesis, Protein Expression, and Purification

SARS-CoV-2 M^pro^ mutants were generated with a QuikChange II Site-Directed Mutagenesis Kit from Agilent (Catalog #200524), using previously created plasmid pE-SUMO-M^pro^ (from strain BetaCoV/Wuhan/WIV04/2019)^26^ as the template. The plasmid produces tag-free M^pro^ protein with no extra residue at either the N or C terminus upon removal of the SUMO tag by SUMO protease digestion.

SARS-CoV-2 M^pro^ mutant proteins were expressed and purified as previously described^26^ with minor modifications. Plasmids were transformed into *E. coli* BL21(DE3) competent cells, bacterial cultures overexpressing the target proteins were grown in LB (Luria–Bertani) medium containing 50 μg/mL of kanamycin at 37 °C, and expression of the target protein was induced at an optical density (A600) of 0.6–0.8 by the addition of isopropyl β-d-1-thiogalactopyranoside (IPTG) to a final concentration of 0.5 mM. The cell culture was incubated at 18 °C for 12–16 h. Bacterial cultures were harvested by centrifugation (8000 × *g*, 10 min, 4 °C) and resuspended in lysis buffer containing 25 mM Tris (pH 8.0), 750 mM NaCl, 2 mM DTT, 0.5 mg/mL lysozyme, 0.5 mM phenylmethylsulfonyl fluoride (PMSF), and 0.02 mg/mL DNase I. Bacterial cells were lysed by alternating sonication (30% amplitude, 1 s on/1 s off) and homogenization using a tissue grinder. The lysed cell suspension was clarified by centrifugation (18 000 × *g*, 30 min, 4 °C), and the supernatant was incubated with Ni-NTA resin for over 2 h at 4 °C on a rotator. The Ni-NTA resin was thoroughly washed with 20 mM imidazole in washing buffer containing 50 mM Tris (pH 8.0), 150 mM NaCl, and 2 mM DTT, and SUMO-M^pro^ protein was eluted with elution buffer containing 50–300 mM imidazole, 50 mM Tris (pH 8.0), 150 mM NaCl, and 2 mM DTT. Fractions containing SUMO-M^pro^ proteins greater than 90% homogeneity were pooled and subjected to dialysis (two times) against a buffer containing 50 mM Tris (pH 8.0), 150 mM NaCl, 2 mM DTT, and 10% glycerol. SUMO protease digestion was carried out at 30 °C for 1 h to remove the SUMO tag. Following digestion, SUMO Protease and SUMO tag were removed by Ni-NTA resin. The purified tag-free SARS-CoV-2 M^pro^ mutant proteins were fast frozen in liquid nitrogen and stored at −80 °C.

### Enzymatic Assays

For measurement of *K*_m_/*V*_max_ of SARS-CoV-2 M^pro^ mutants, proteolytic reactions were carried out with optimized concentrations (Table S4) of the mutant proteins and series concentrations of FRET substrate (0, 1.56, 3.13, 6.25, 12.5, 25, 50, 100, and 200 μM) in 100 μL of reaction buffer containing 20 mM HEPES (pH 6.5), 120 mM NaCl, 0.4 mM EDTA, 4 mM DTT, and 20% glycerol at 30 °C in a BioTek Cytation 5 imaging reader (Agilent) with filters for excitation at 360/40 nm and emission at 460/40 nm. The maximum concentration of FRET substrate was set at 200 μM to avoid inner filter effect, as concentrations below 200 μM have linear dependence of fluorescence intensity. Reactions were monitored every 90 s, and the initial velocity of the proteolytic activity was calculated by linear regression for the first 15 min of the kinetic progress curves. The initial velocity was plotted against the FRET substrate concentrations using the classic Michaelis–Menten equation (*Y* = *V*_max_*X/(*K*_m_ + *X*), *X* = substrate concentration; *Y* = enzyme velocity) in Prism 8 software.

For IC_50_ measurements, optimized concentrations (Table S4) of the mutant proteins were incubated with series concentrations of GC-376, PF-00835231, or nirmatrelvir (PF-07321332) in 100 μL of reaction buffer at 30 °C for 15 min, and the reaction was initiated by adding 10 μM M^pro^ FRET substrate. The reaction was monitored for 1 h, and the initial velocity was calculated for the first 15 min by linear regression. The IC_50_ was determined by plotting the initial velocity against various concentrations of the compounds using the following equation: (*Y* = 100/(1 + 10̂((LogIC_50_ – *X*)*HillSlope)), *X* = log of inhibitor concentration; *Y* = normalized enzyme velocity) in Prism 8 software.

For *K*_i_ measurements, optimized concentrations (Table S4) of the mutant proteins were added to 20 μM M^pro^ FRET substrate with various concentrations of GC-376, PF-00835231, or nirmatrelvir (PF-07321332) in 200 μL of reaction buffer at 30 °C to initiate the proteolytic reaction. The reaction was monitored for 2 h, and the initial velocity was calculated for the first 90 min by linear regression. The *K*_i_ was calculated using the Morrison equation for tight binding (*Y* = *V*_0_*(1 – ((((*E*_t_ + *X* + (*K*_i_*(1 + (*S*/*K*_m_)))) – (((*E*_t_ + *X* + (*K*_i_*(1 + (*S*/*K*_m_))))^^2^) – 4*E_t_*X)^^0.5^))/(2*E_t_))), *X* = inhibitor concentration; *Y* = enzyme velocity; *E*_t_ = enzyme concentration; *V*_0_ = enzyme velocity in the absence of inhibitor) by plotting the initial velocity against various concentrations of the compounds using Morrison plot (tight binding) in Prism 8 software.

All reported values (*K*_m_, *V*_max_, IC_50_, and *K*_i_) are the average of two replicates ± standard error (SE) with a 95% confidence interval calculated as SE = (upper limit – lower limit)/3.92.

### Differential Scanning Fluorimetry (DSF)

The binding of nirmatrelvir (PF-07321332) to SARS-CoV-2 mutant proteins was monitored by differential scanning fluorimetry (DSF) using a QuantStudio 5 Real-Time PCR System (Thermo Fisher) as previously described^39,40^ with minor modifications. Briefly, 6 μM of WT or the mutant M^pro^ proteins was mixed with serial concentrations of nirmatrelvir (0, 0.2, 0.6, 2, 6, 20, 60, 200 μM) in 50 μL of reaction buffer in a 96-well PCR plate, and the plate was incubated at 30 °C for 1 h. After incubation, 1× SYPRO orange (Thermo Fisher) was added, and the fluorescence signal was recorded under a temperature gradient ranging from 20 to 95 °C (incremental steps of 0.05 °C s^–1^). The melting temperature (*T*_m_) was calculated as the mid log of the transition phase from the native to the denatured protein using a Boltzmann model in Protein Thermal Shift Software v1.3. Δ*T*_m_ was calculated by subtracting the reference melting temperature of proteins in the presence of DMSO from the *T*_m_ in the presence of compounds. The reported Δ*T*_m_ values were averages of two replicates. Curve fitting was performed using log (inhibitor) vs Δ*T*_m_ – variable slope in Prism (v8) software.

### Generation of Nsp5 Mutant Viruses

The present work with infectious SARS-CoV-2 was approved by the Institutional Biosafety Committee (IBC#21–22) and carried out in a fully certified Biosafety level-3 laboratory at Oklahoma State University. To generate recombinant Nsp5^S144A^, Nsp5^S144M^, Nsp5^E166Q^, Nsp5^H172Q^, and Nsp5^H172Y^ mutant viruses, corresponding nucleotide substitutions were introduced into the SARS-CoV-2 infectious cDNA subclone plasmid using a Q5 site-directed mutagenesis kit (NEB, E0554S) and then verified by sequencing of the plasmid. Virus recovery was conducted as described previously.^30^ Briefly, viral cDNA fragments were ligated in an equal molar ratio to assemble a full-length genomic cDNA with T4 DNA ligase (NEB, M0202L). The ligated full-length cDNA and a SARS-CoV-2-N plasmid were used for *in vitro* transcription using the T7 mMESSAGE mMACHINE T7 transcription kit (ThermoFisher, AM1344). The transcribed viral RNA and N gene sgRNA were subsequently electroporated into Vero-TA cells. These cells were maintained in DMEM containing 2% FBS at 37 °C. Culture supernatants were collected at the time when the cytopathic effect was evident. All harvested viral stocks were titrated in Vero-TA cells and subjected to sequencing of the Nsp5 coding region to validate the genotypes.

### Growth Kinetics and Plaque Assay

To determine viral growth kinetics, Vero-TA cells were seeded in 12- or 24- well plates a day prior to infection and inoculated with the designated virus at a multiplicity of infection (MOI) of 0.01 for 1 h at 37 °C. After 1 h of incubation, the viral inoculum was removed and replaced with fresh DMEM containing 2% FBS. The culture supernatants were collected at the indicated time points and titrated on Vero-TA cells using a plaque assay. For plaque assay, Vero-TA 2 cells were seeded in 6- or 12-well plates a day prior to infection. Each viral stock supernatant was serially diluted and inoculated onto the Vero-TA cells. After 1 h of incubation at 37 °C, the inoculum was removed, and cells were subsequently overlaid with a 1.2% 2 × DMEM–agarose mixture. After 48 h, cells were fixed using 4% formaldehyde for 1 h and stained using 0.1% crystal violet solution after removal of the agarose overlay. Plaques were photographed and counted, and titers were calculated.

### Antiviral Plaque Assay

The antiviral plaque assay was performed similarly as we described previously.^25^ Briefly, nirmatrelvir dissolved in DMSO was serially diluted in DMEM as a 6-pt dose–response with threefold dilutions between test concentrations, starting at 10 μM final concentration. Vero-TA cells in 12-well plates were incubated with approximately 50 PFU per well of each virus for 1 h. After incubation, the inoculum was removed, and 1 mL of DMEM–1.2% Avicel (FMC polymers) mixture containing serially diluted nirmatrelvir and 2uM P-glycoprotein inhibitor, CP-100356, was added to each well. After 48 h of incubation at 37 °C, the DMEM–Avicel mixture was removed, and the cells were stained using 0.1% crystal violet solution. Plates were photographed and measured for the area of cells affected by infection using ImageJ.

### Virus Passage Assay

Vero-TA cells (3 × 10^5^ cell per well) in 12-well plates were infected with passage 0 (P0) recombinant Nsp5 mutant viruses at a MOI of 0.01. For each passage, culture supernatant was collected 24 h postinfection, and 3 uL of it with an estimated 3 × 10^4^ PFU was used to infect the next well of cells. This passage experiment was carried out three times, and the collected culture supernatants were extracted for RNA using Trizol (Thermo Fisher), followed by cDNA synthesis using LunaScript RT SuperMix (NEB). A viral genomic fragment (9642–11068 nts) containing Nsp5 gene was PCR amplified with primers (Forward primer: TTCAGTGGATGGTTATGTTCACACCT, Reverse primer: AGACCATTGAGTACTCTGGACT) using Q5 Hot Start High-Fidelity 2X Master Mix (NEB). The PCR products were Sanger sequenced, and the percentage of each genotype was determined by analyzing the chromatographic values of each nucleotide in the sequencing trace files.

### M^pro^ Crystallization and Structure Determination

SARS-CoV-2 M^pro^ was diluted to 5 mg/mL in protein buffer (50 mM Tris pH 7.0, 150 mM NaCl, 4 mM DTT). To prepare inhibitor complexes, protein was incubated overnight at 4 °C with either 2 mM GC-376 or 2 mM nirmatrelvir. Proteins with nirmatrelvir were supplemented with 4% DMSO to enhance solubility of the compound, and precipitation during incubation was removed by centrifugation prior to crystallization. Since GC-376 is water-soluble, no precipitation was observed, and centrifugation was not necessary. Crystals were grown by mixing 1.5 μL of the protein solution with 1.5 μL of the precipitant solution in a hanging-drop vapor-diffusion apparatus. Three precipitant conditions were used for crystal growth: 1. 25% PEG 3350, 0.1 M K/Na tartrate, and 0.005 M MgCl_2_; 2. 0.2 M NaCl, 10% 1,6-hexanediol, and 20% PEG MME 2K; 3. 0.1 M MgCl_2_, 20% PEG 3350, 10% 1,6-hexanediol, 0.1 M HEPES pH 7.5, and 0.1 M Li_2_SO_4_. Crystals were transferred to a cryoprotectant solution and flash-frozen in liquid nitrogen. Cryoprotectant solution was varied based on the crystallization condition as follows: 1. 27.5% PEG 3350, 0.1 M K/Na tartrate, 0.005 M MgCl_2_, and 15% glycerol; 2. 0.2 M NaCl, 10% 1,6-hexanediol, 20% PEG MME 2K, and 20% glycerol; 3. 0.1 M MgCl_2_, 15% PEG 3350, 10% 1,6-hexanediol, 0.1 M HEPES pH 7.5, 0.1 M LiSO_4_, and 20% glycerol.

X-ray diffraction data were collected at the Southeast Regional Collaborative Access Team (SER-CAT) 22-ID and 22-BM beamlines at the Advanced Photon Source (APS) in Argonne, IL, and processed with HKL2000 and CCP4. PHASER was used for molecular replacement using a previously solved SARS-CoV-2 M^pro^ structure (PDB ID: 7LYH) as a reference model. The CCP4 suite,^41^ Coot,^42^ and the PDB REDO server (pdb-redo.edu)^43^ were used to complete the model building and refinement. The PyMOL Molecular Graphics System (Schrödinger, LLC) was used to generate all images. The X-ray crystal structures have been deposited into the Protein Data Bank with accession codes 8D4J (H172Y apo), 8D4K (H172Y-GC376), 8D4L (S144A), 8D4M (S144A-GC376), 8D4N (E166Q apo), 8DFN (H164N apo), 8DD1 (H164N-GC376), 8DFE (S144L apo), 8DD9 (S144L-GC376), 8DGB (Q192T-GC376), and 8DCZ (M165Y-nirmatrelvir).

## Supporting Information Available

SDS-PAGE and thermal shift assay results; determination of *K_i_* values; plaque formation; X-ray crystal structures, enzymatic characterization, drug inhibition, and PDB code of SARS-CoV-2 Mpro mutant; X-ray data collection and refinement statistics; protein concentrations used (Figures S1–S5 and Tables S1–S4) (PDF)
