# Supplementary material for: Naturally Occurring Mutations of SARS-CoV-2 Main Protease Confer Drug Resistance to Nirmatrelvir
Source: ACS Cent Sci. 2023 Jul 24;9(8):1658–69. doi: 10.1021/acscentsci.3c00538 (PMC10451032; doi:10.1021/acscentsci.3c00538)
Supplement: Supplementary file 1 — oc3c00538_si_001.pdf [file oc3c00538_si_001.pdf]

Supplementary Materials for

Naturally occurring mutations of SARS-CoV-2 main protease confer drug resistance to nirmatrelvir

Yanmei Hu,[1,†] Eric M. Lewandowski,[2,†] Haozhou Tan,[1,†] Xiaoming Zhang,[3,4] Ryan T. Morgan,[2] Xiujun Zhang,[2] Lian M. C. Jacobs,[2] Shane G. Butler,[2] Maura V. Gongora,[2] John Choy,[5] Xufang Deng,[3,4,*] Yu Chen,[2,*] Jun Wang[1,*]

[1]Department of Medicinal Chemistry, Ernest Mario School of Pharmacy, Rutgers, the State University of New Jersey, NJ, 08854, United States

[2]Department of Molecular Medicine, Morsani College of Medicine, University of South Florida, Tampa, FL, 33612, United States

[3]Department Physiological Sciences, College of Veterinary Medicine, Oklahoma State University, Stillwater, OK, 74078, United States

[4]Oklahoma Center for Respiratory and Infectious Diseases, Oklahoma State University, Stillwater, OK, 74078, United States

[5]Department Biology, School of Arts and Sciences, the Catholic University of America, Washington DC, 20064, United States

[†]These authors contributed equally to this work.

*Corresponding authors:

Jun Wang, Tel: 848-445-6488, email: junwang@pharmacy.rutgers.edu

Yu Chen, Tel: 813-974-7809, email: ychen1@usf.edu

Xufang Deng, Tel: 405-744-2158, email: xufang.deng@okstate.edu

This file includes:

Supplementary Materials

Figure S1. Sodium dodecyl sulfate-polyacrylamide gel electrophoresis (SDS-PAGE) analysis of purified SARS-CoV-2 M^{pro} WT and mutant proteins.

Figure S2. Thermal shift assay results of M^{pro} mutants.

Figure S3. Determination of K_i values for GC-376, PF-00835231 or Nirmatrelvir (PF-07321332) against SARS-CoV-2 WT and mutant proteins in FRET assay.

Figure S4. Plaque formation of passaged recombinant Nsp5 mutants.

Figure S5. X-ray crystal structures of H164N mutant.

Table S1. Enzymatic characterization, drug inhibition, and PDB code of SARS-CoV-2 M^{pro} mutants.

Table S2. Thermal shift assay results of nirmatrelvir with M^{pro} mutants.

Table S3. X-ray Data Collection and Refinement Statistics.

Table S4. Protein concentrations used for the K_m/V_{max}, IC_{50} and K_i measurements.

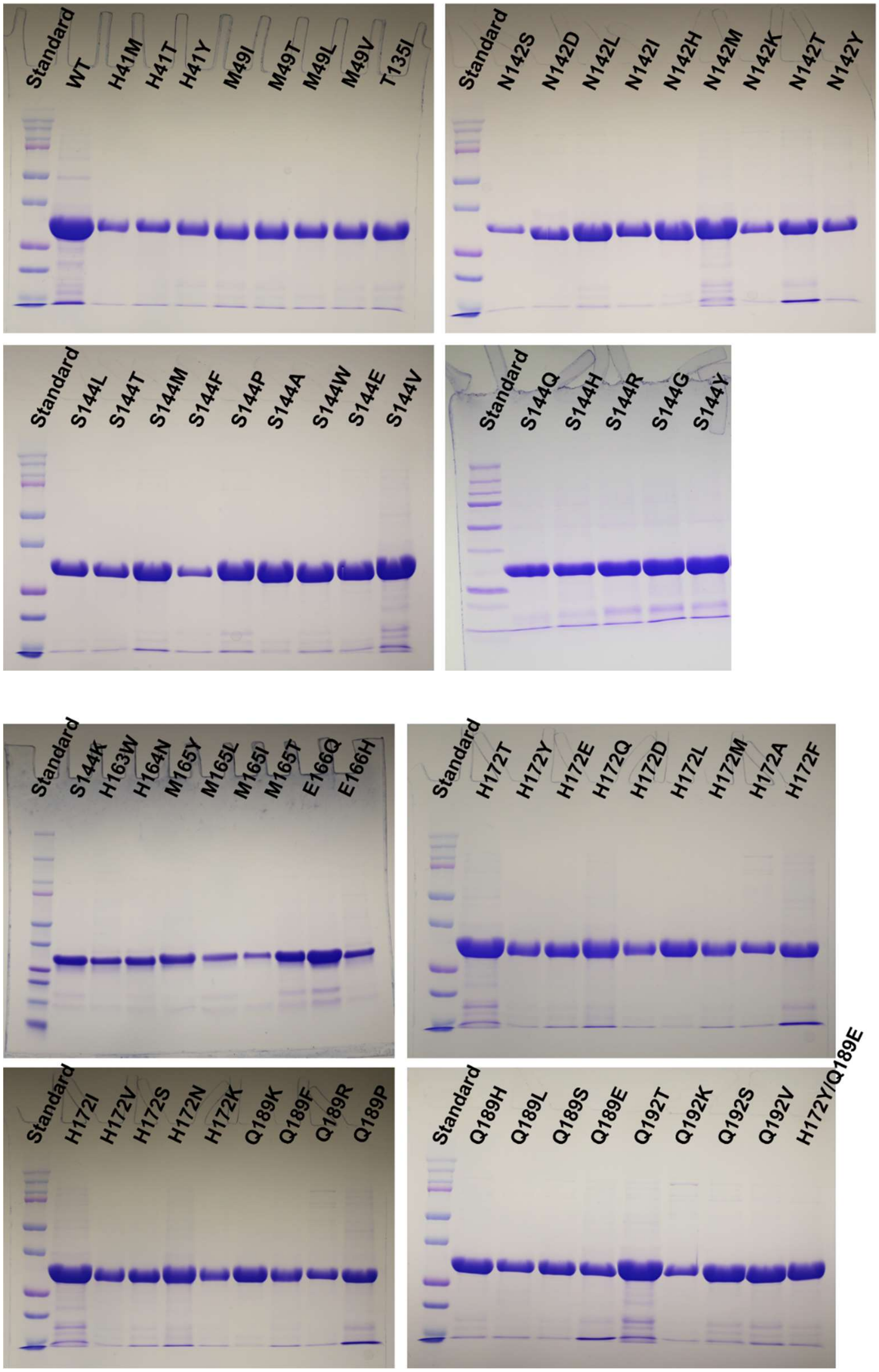
Standard
WT
H41M
H41T
H41Y
M49I
M49T
M49L
M49V
T135I
Standard
N142S
N142D
N142L
N142I
N142H
N142M
N142K
N142T
N142Y
Standard
S144L
S144T
S144M
S144F
S144P
S144A
S144W
S144E
S144V
Standard
S144Q
S144H
S144R
S144G
S144Y
Standard
S144K
H163W
H164N
M165Y
M165L
M165I
M165T
E166Q
E166H
Standard
H172T
H172Y
H172E
H172Q
H172D
H172L
H172M
H172A
H172F
Standard
H172I
H172V
H172S
H172N
H172K
Q189K
Q189F
Q189R
Q189P
Standard
Q189H
Q189L
Q189S
Q189E
Q192T
Q192K
Q192S
Q192V
H172Y/Q189E

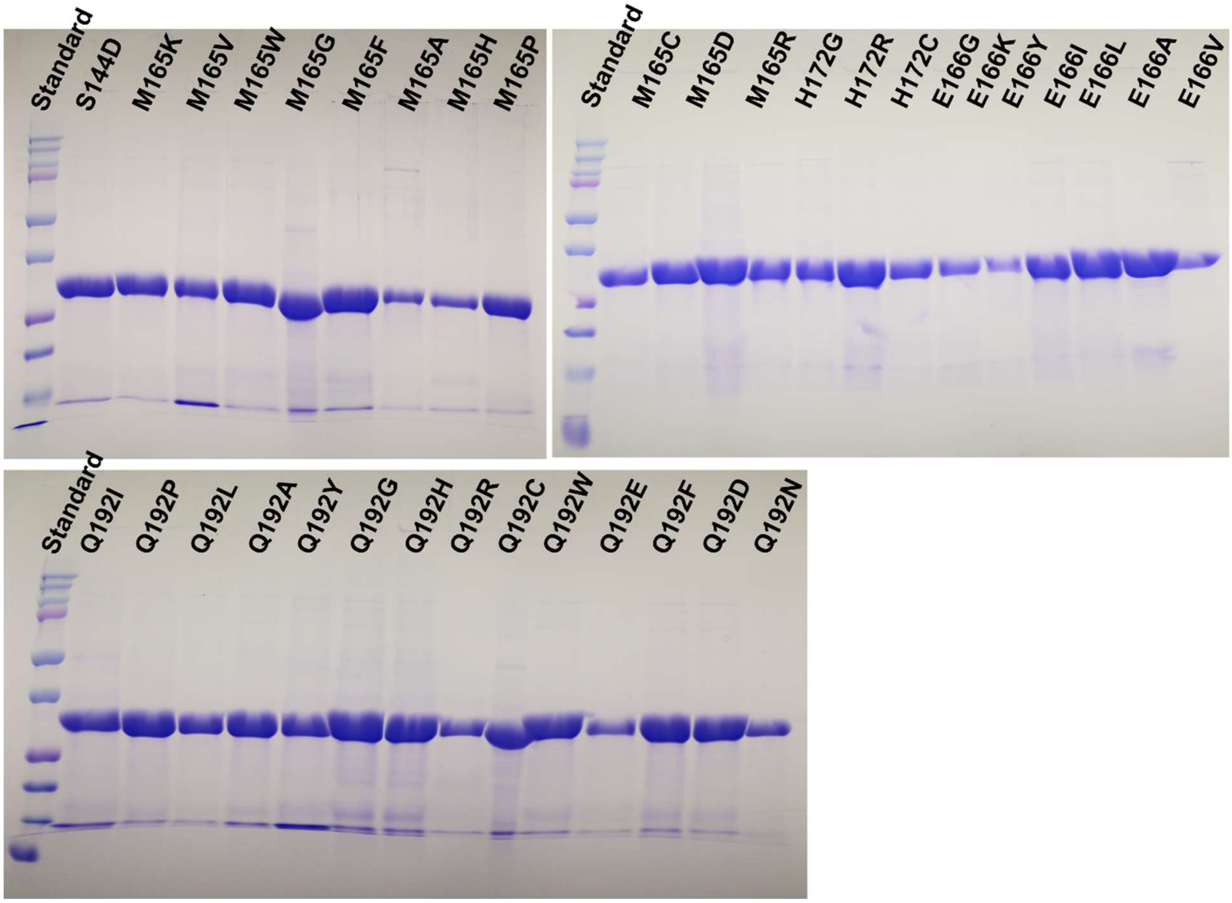

Figure S1. Sodium dodecyl sulfate-polyacrylamide gel electrophoresis (SDS-PAGE) analysis of purified SARS-CoV-2 M^{pro} WT and mutant proteins. 10 µl of purified proteins were analyzed on 15% SDS-PAGE gel and the protein bands were visualized by staining with Coomassie blue. Protein standard (10-250 kD) was purchased from Bio-Rad, Cat #1610374.

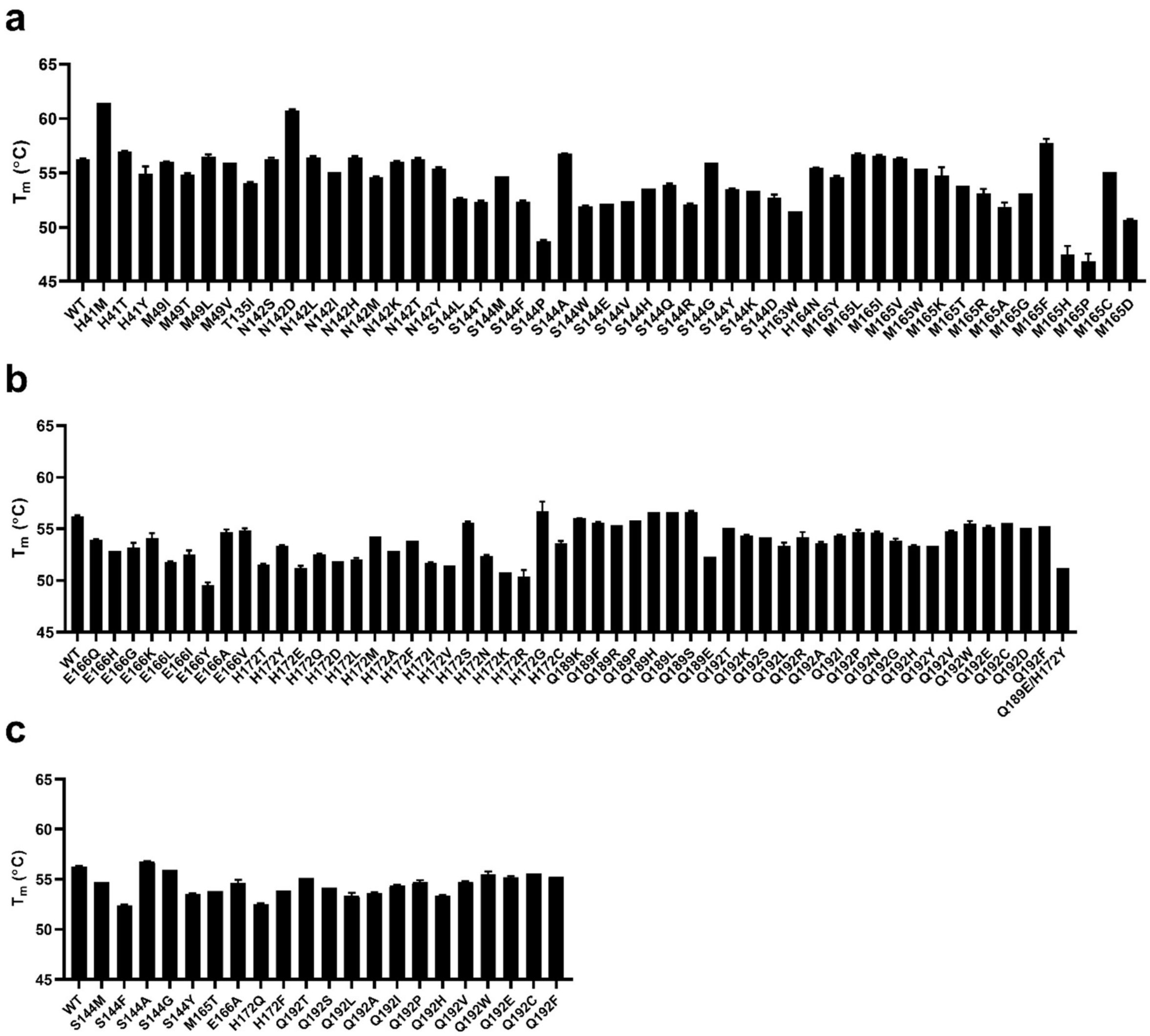

Figure S2. **Thermal shift assay results of M^{pro} mutants.**

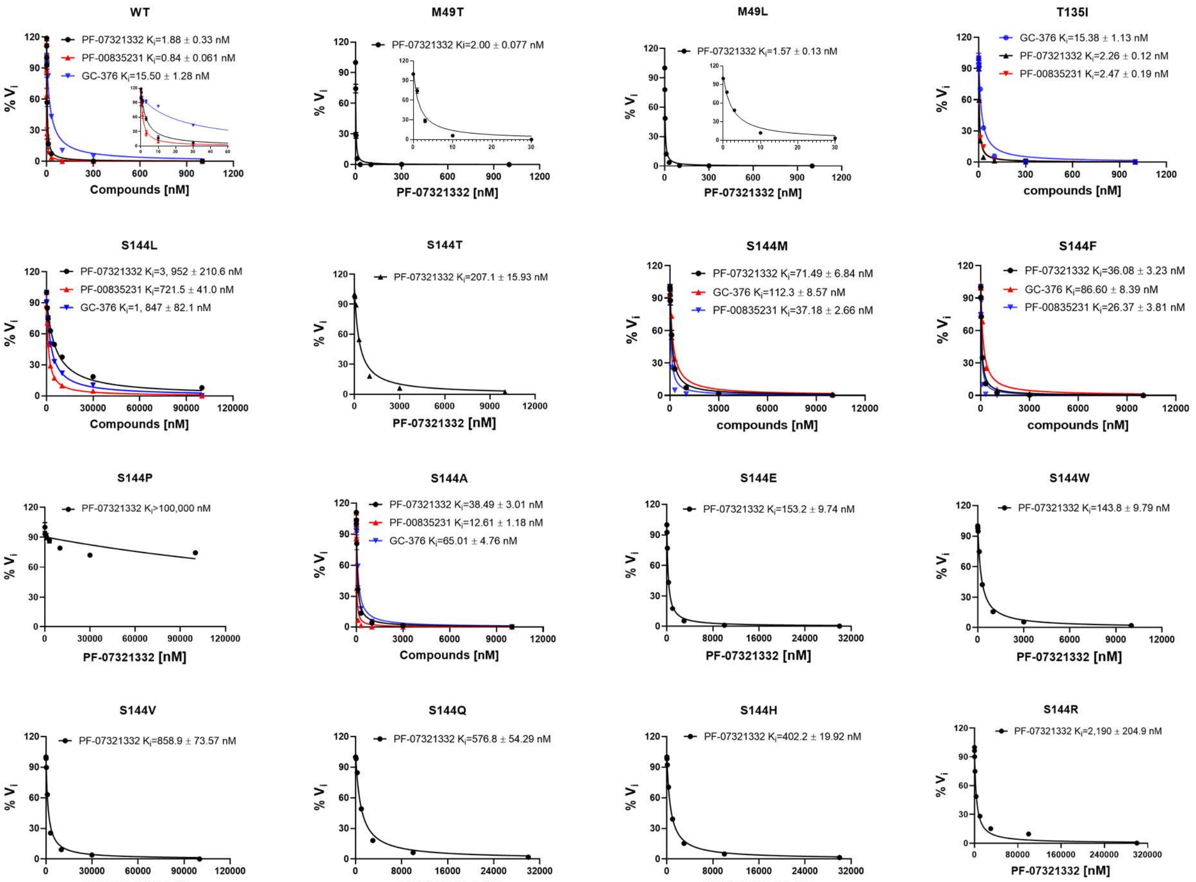
WT
PF-07321332 Ki=1.88 ± 0.33 nM
PF-00835231 Ki=0.84 ± 0.061 nM
GC-376 Ki=15.50 ± 1.28 nM
Compounds [nM]
M49T
PF-07321332 Ki=2.00 ± 0.077 nM
M49L
PF-07321332 Ki=1.57 ± 0.13 nM
T135I
GC-376 Ki=15.38 ± 1.13 nM
PF-07321332 Ki=2.26 ± 0.12 nM
PF-00835231 Ki=2.47 ± 0.19 nM
compounds [nM]
S144L
PF-07321332 Ki=3, 952 ± 210.6 nM
PF-00835231 Ki=721.5 ± 41.0 nM
GC-376 Ki=1, 847 ± 82.1 nM
S144T
PF-07321332 Ki=207.1 ± 15.93 nM
S144M
PF-07321332 Ki=71.49 ± 6.84 nM
GC-376 Ki=112.3 ± 8.57 nM
PF-00835231 Ki=37.18 ± 2.66 nM
S144F
PF-07321332 Ki=36.08 ± 3.23 nM
GC-376 Ki=86.60 ± 8.39 nM
PF-00835231 Ki=26.37 ± 3.81 nM
S144P
PF-07321332 Ki>100,000 nM
S144A
PF-07321332 Ki=38.49 ± 3.01 nM
PF-00835231 Ki=12.61 ± 1.18 nM
GC-376 Ki=65.01 ± 4.76 nM
S144E
PF-07321332 Ki=153.2 ± 9.74 nM
S144W
PF-07321332 Ki=143.8 ± 9.79 nM
S144V
PF-07321332 Ki=858.9 ± 73.57 nM
S144Q
PF-07321332 Ki=576.8 ± 54.29 nM
S144H
PF-07321332 Ki=402.2 ± 19.92 nM
S144R
PF-07321332 Ki=2,190 ± 204.9 nM
% Vi
PF-07321332 [nM]

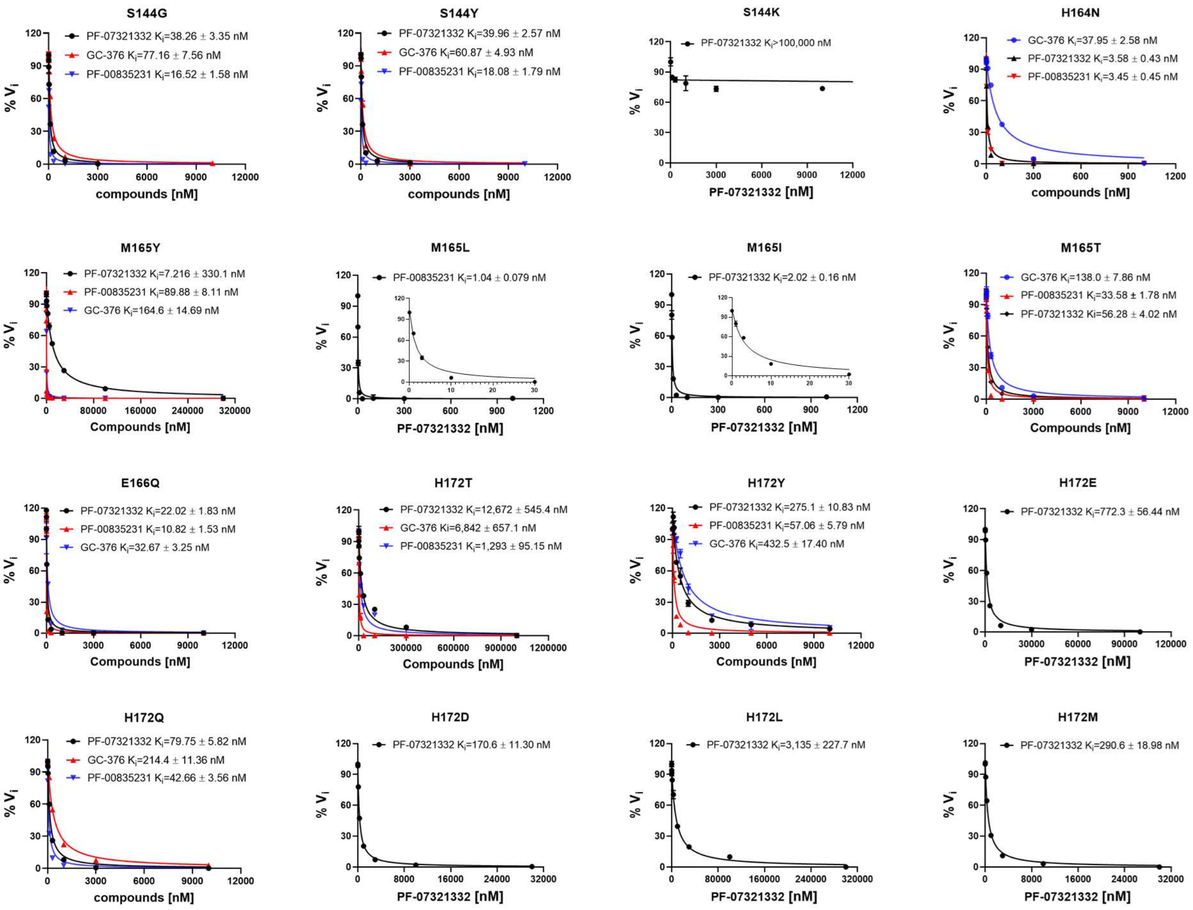
S144G
PF-07321332 K_i=38.26 ± 3.35 nM
GC-376 K_i=77.16 ± 7.56 nM
PF-00835231 K_i=16.52 ± 1.58 nM
compounds [nM]
S144Y
PF-07321332 K_i=39.96 ± 2.57 nM
GC-376 K_i=60.87 ± 4.93 nM
PF-00835231 K_i=18.08 ± 1.79 nM
compounds [nM]
S144K
PF-07321332 K_i>100,000 nM
PF-07321332 [nM]
H164N
GC-376 K_i=37.95 ± 2.58 nM
PF-07321332 K_i=3.58 ± 0.43 nM
PF-00835231 K_i=3.45 ± 0.45 nM
compounds [nM]
M165Y
PF-07321332 K_i=7.216 ± 330.1 nM
PF-00835231 K_i=89.88 ± 8.11 nM
GC-376 K_i=164.6 ± 14.69 nM
Compounds [nM]
M165L
PF-00835231 K_i=1.04 ± 0.079 nM
PF-07321332 [nM]
M165I
PF-07321332 K_i=2.02 ± 0.16 nM
PF-07321332 [nM]
M165T
GC-376 K_i=138.0 ± 7.86 nM
PF-00835231 K_i=33.58 ± 1.78 nM
PF-07321332 Ki=56.28 ± 4.02 nM
Compounds [nM]
E166Q
PF-07321332 K_i=22.02 ± 1.83 nM
PF-00835231 K_i=10.82 ± 1.53 nM
GC-376 K_i=32.67 ± 3.25 nM
Compounds [nM]
H172T
PF-07321332 K_i=12,672 ± 545.4 nM
GC-376 Ki=6,842 ± 657.1 nM
PF-00835231 K_i=1,293 ± 95.15 nM
Compounds [nM]
H172Y
PF-07321332 K_i=275.1 ± 10.83 nM
PF-00835231 K_i=57.06 ± 5.79 nM
GC-376 K_i=432.5 ± 17.40 nM
Compounds [nM]
H172E
PF-07321332 K_i=772.3 ± 56.44 nM
PF-07321332 [nM]
H172Q
PF-07321332 K_i=79.75 ± 5.82 nM
GC-376 K_i=214.4 ± 11.36 nM
PF-00835231 K_i=42.66 ± 3.56 nM
compounds [nM]
H172D
PF-07321332 K_i=170.6 ± 11.30 nM
PF-07321332 [nM]
H172L
PF-07321332 K_i=3,135 ± 227.7 nM
PF-07321332 [nM]
H172M
PF-07321332 K_i=290.6 ± 18.98 nM
PF-07321332 [nM]
% V_i

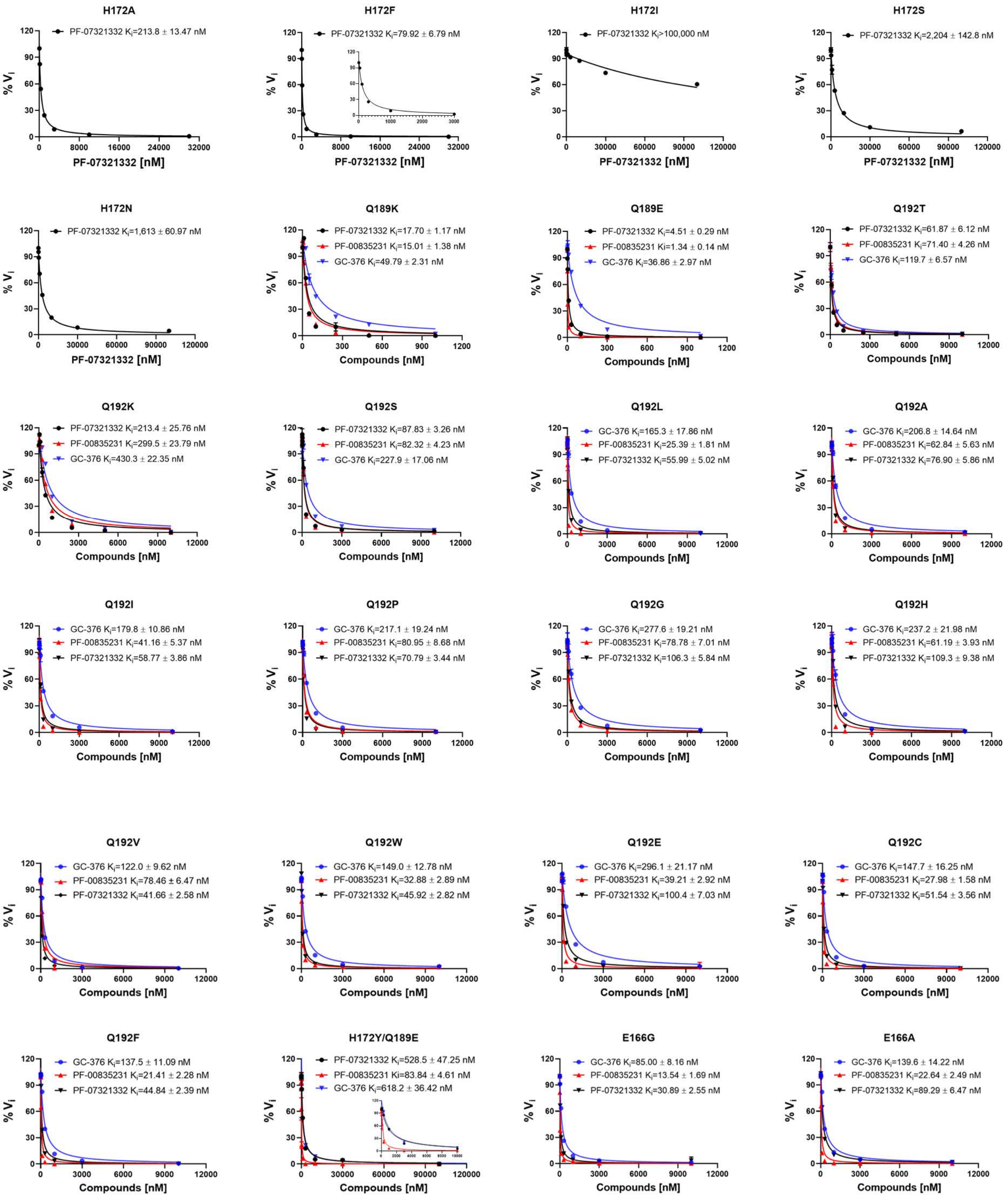

Figure S3. Determination of K_i values for GC-376, PF-00835231 or Nirmatrelvir (PF-07321332) against SARS-CoV-2 WT and mutant proteins in FRET assay. Curves were generated by fitting the initial velocity against various concentrations of the

compounds using Morrison plot (tight binding) in Prism 8 software. The results were the average of duplicates.

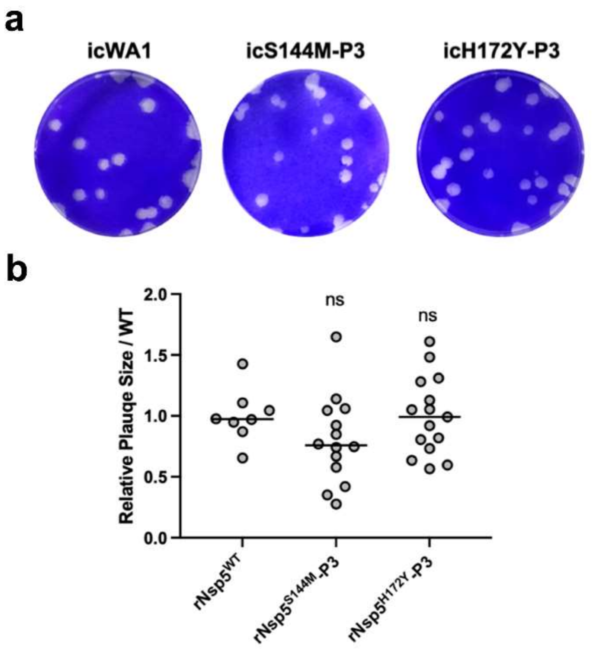

Figure S4. Plaque formation of passaged recombinant Nsp5 mutants. a Plaque formation by rNsp5^{WT}, rNsp^{S144M}-P3, and rNsp5^{H172Y}-P3 mutant viruses. **b** Plaque sizes were measured for each virus using ImageJ. Relative plaque sizes over WT are presented as mean ± SEM. Statistical significance of the size differences of each virus vs. rNsp5^{WT} virus was determined using the unpaired Kolmogorov-Smirnov test in Prism 9. ns: no significant.

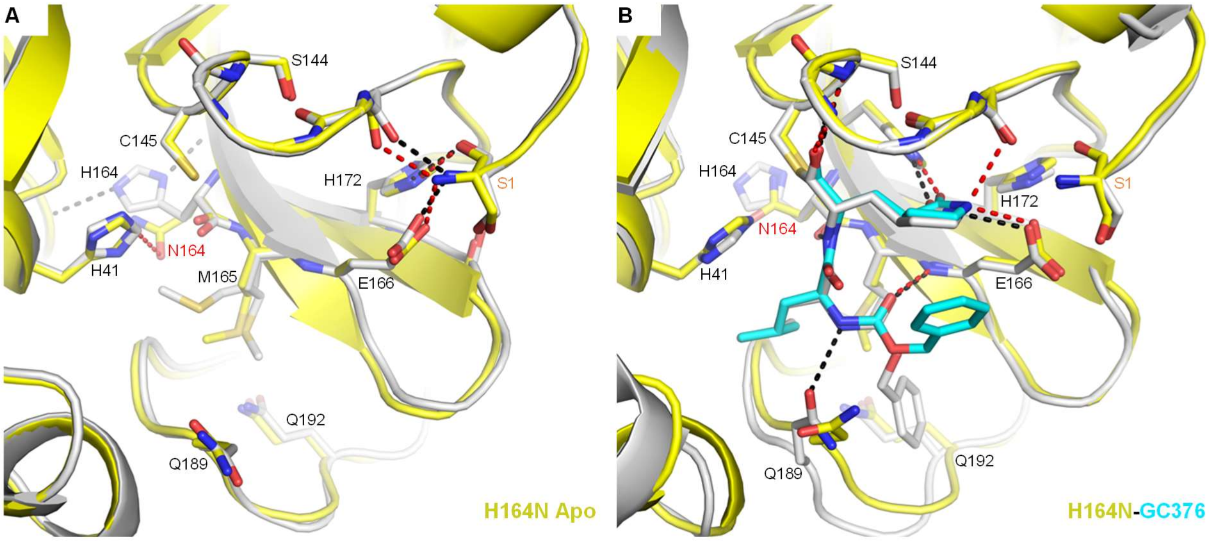

Figure S5. X-ray crystal structures of H164N mutant. a Apo M^{pro} WT (white, PDB 7JP1) aligned with apo M^{pro} H164N (yellow, PDB 8DFN). **b** M^{pro} WT GC376 complex (white, PDB 6WTT) aligned with M^{pro} H164N GC376 complex (yellow, PDB 8DD1). WT hydrogen bonds are shown as black dashes, and mutant hydrogen bonds are shown as red dashes. GC-376 is shown in white for the WT structure and cyan for the mutant structure. Mutation is indicated with red text. Ser1 from an adjacent monomer is indicated with orange text.

Table S1. Enzymatic characterization, drug inhibition, and PDB code of SARS-CoV-2 M^{pro} mutants.

**Resistant mutants identified from the GISAID SARS-CoV-2 sequence analysis**						
M^{pro} mutants	Occurrence[a]	k_{cat}, V_{max}, K_m, k_{cat}/K_m	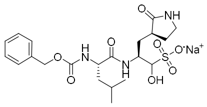 GC-376	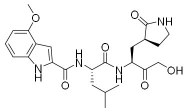 PF-00835231	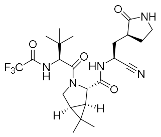 Nirmatrelvir (PF-07321332)	PDB code
WT		K_m = 35.36 ± 2.41 μM<br>V_{max} = 38.97 ± 0.91 nM/s<br>k_{cat} = 0.39 S^{-1}<br>k_{cat}/K_m = 11,000 $S^{-1}M^{-1}$	IC_{50} = 40.25 ± 1.61 nM<br>K_i = 15.50 ± 1.28 nM	IC_{50} = 15.09 ± 0.80 nM<br>K_i = 0.84 ± 0.061 nM	IC_{50} = 26.03 ± 1.65 nM<br>K_i =1.88 ± 0.33 nM	
H41M	84	Enzymatically inactive				
H41T	27	Enzymatically inactive				
H41Y	19	Enzymatically inactive				
M49I	2,080	K_m = 21.43 ± 3.29 μM<br>V_{max} = 19.90 ± 0.94 nM/s<br>k_{cat} = 0.3980 S^{-1}<br>k_{cat}/K_m = 18,572 $S^{-1}M^{-1}$	IC_{50} = 41.55 ± 0.75 nM	IC_{50} = 17.84 ± 0.64 nM	IC_{50} = 26.54 ± 1.05 nM	
M49T	78	K_m = 21.58 ± 2.61 μM<br>V_{max} = 14.47 ± 1.08 nM/s<br>k_{cat} = 0.2315 S^{-1}<br>k_{cat}/K_m = 10,728 $S^{-1}M^{-1}$			IC_{50} = 15.76 ± 0.69 nM<br>K_i = 2.00 ± 0.08 nM	
M49L	73	K_m = 29.02 ± 2.42 μM<br>V_{max} = 34.77 ± 1.93 nM/s<br>k_{cat} = 0.5563 S^{-1}<br>k_{cat}/K_m = 19,170 $S^{-1}M^{-1}$			IC_{50} = 18.50 ± 0.69 nM<br>K_i = 1.57 ± 0.13 nM	
M49V	55	K_m = 32.35 ± 1.65 μM<br>V_{max} = 23.75 ± 0.92 nM/s<br>k_{cat} = 0.1900 S^{-1}<br>k_{cat}/K_m = 5,873 $S^{-1}M^{-1}$			IC_{50} = 23.48 ± 0.39 nM	
M49 deletion	29	K_m = 30.87 ± 3.16 μM<br>V_{max} = 17.45 ± 0.60 nM/s<br>k_{cat} = 0.1745 S^{-1}<br>k_{cat}/K_m = 5,653 $S^{-1}M^{-1}$			IC_{50} = 32.99 ± 1.94 nM<br>K_i = 8.02 ± 0.82 nM	
T135I	1,342	K_m = 27.08 ± 2.76 μM<br>V_{max} = 29.28 ± 0.97 nM/s<br>k_{cat} = 0.2928 S^{-1}<br>k_{cat}/K_m = 10,812 $S^{-1}M^{-1}$	IC_{50} = 42.12 ± 3.41 nM<br>K_i = 15.38 ± 1.13 nM	IC_{50} = 15.63 ± 1.05 nM<br>K_i = 2.47 ± 0.19 nM	IC_{50} = 21.86 ± 0.85 nM<br>K_i = 2.26 ± 0.12 nM	
T135 deletion	40	K_m = 41.78 ± 3.99 μM<br>V_{max} = 13.94 ± 0.49 nM/s<br>k_{cat} = 0.0028 S^{-1}<br>k_{cat}/K_m = 67 $S^{-1}M^{-1}$				

N142S	142	K_m = 22.24 ± 3.09 μM<br>V_{max} = 26.07 ± 1.13 nM/s<br>k_{cat} = 0.2607 S^{-1}<br>k_{cat}/K_m = 11,722 $S^{-1}M^{-1}$	IC_{50} = 39.92 ± 1.36 nM	IC_{50} = 17.64 ± 1.18 nM	IC_{50} = 25.97 ± 1.14 nM	
N142D	76	K_m = 29.03 ± 2.98 μM<br>V_{max} = 9.91 ± 0.34 nM/s<br>k_{cat} = 0.0793 S^{-1}<br>k_{cat}/K_m = 2,731 $S^{-1}M^{-1}$	IC_{50} = 37.90 ± 1.67 nM	IC_{50} = 18.23 ± 0.49 nM	IC_{50} = 24.30 ± 1.46 nM	
N142L	34	K_m = 25.93 ± 2.51 μM<br>V_{max} = 24.94 ± 1.57 nM/s<br>k_{cat} = 0.3990 S^{-1}<br>k_{cat}/K_m = 15,389 $S^{-1}M^{-1}$			IC_{50} = 15.29 ± 0.42 nM	
N142I	18	K_m = 31.02 ± 5.85 μM<br>V_{max} = 32.86 ± 2.10 nM/s<br>k_{cat} = 0.5258 S^{-1}<br>k_{cat}/K_m = 16,949 $S^{-1}M^{-1}$			IC_{50} = 17.11 ± 0.82 nM	
N142H	13	K_m = 41.81 ± 5.14 μM<br>V_{max} = 29.84 ± 1.35 nM/s<br>k_{cat} = 0.2387 S^{-1}<br>k_{cat}/K_m = 5,710 $S^{-1}M^{-1}$			IC_{50} = 19.65 ± 0.32 nM	
N142M	9	K_m = 10.02 ± 3.40 μM<br>V_{max} = 10.88 ± 0.96 nM/s<br>k_{cat} = 0.1741 S^{-1}<br>k_{cat}/K_m = 17,373 $S^{-1}M^{-1}$			IC_{50} = 19.01 ± 0.62 nM	
N142K	8	K_m = 43.57 ± 4.72 μM<br>V_{max} = 20.14 ± 0.81 nM/s<br>k_{cat} = 0.3222 S^{-1}<br>k_{cat}/K_m = 7,396 $S^{-1}M^{-1}$			IC_{50} = 13.93 ± 0.39 nM	
N142T	4	K_m = 21.99 ± 4.78 μM<br>V_{max} = 13.66 ± 0.92 nM/s<br>k_{cat} = 0.2186 S^{-1}<br>k_{cat}/K_m = 9,939 $S^{-1}M^{-1}$			IC_{50} = 18.73 ± 0.96 nM	
N142Y	4	K_m = 20.84 ± 2.45 μM<br>V_{max} = 11.95 ± 0.86 nM/s<br>k_{cat} = 0.1912 S^{-1}<br>k_{cat}/K_m = 9,175 $S^{-1}M^{-1}$			IC_{50} = 14.90 ± 1.26 nM	
N142 deletion	91	K_m = 31.35 ± 3.46 μM<br>V_{max} = 10.86 ± 0.032 nM/s<br>k_{cat} = 0.002172 S^{-1}<br>k_{cat}/K_m = 69 $S^{-1}M^{-1}$				
S144L	52	K_m = 51.29 ± 3.20 μM<br>V_{max} = 6.11 ± 0.15 nM/s<br>k_{cat} = 0.0031 S^{-1}<br>k_{cat}/K_m = 60 $S^{-1}M^{-1}$	IC_{50} = 1,812 ± 86.1 nM<br>K_i = 1,847 ± 82.1 nM	IC_{50} = 716.7 ± 42.5 nM<br>K_i = 721.5 ± 41.0 nM	IC_{50} = 5,364 ± 498.6 nM<br>K_i = 3,952 ± 210.6 nM	Apo: 8DFE<br>GC-376: 8DD9
S144T	17	K_m = 44.31 ± 2.92 μM<br>V_{max} = 16.85 ± 0.42 nM/s<br>k_{cat} = 0.0225 S^{-1}<br>k_{cat}/K_m = 507 $S^{-1}M^{-1}$			IC_{50} = 353.7 ± 14.36 nM<br>K_i = 207.1 ± 15.93 nM	
S144M	16	K_m = 51.85 ± 3.37 μM<br>V_{max} = 28.36 ± 0.72 nM/s<br>k_{cat} = 0.0709 S^{-1}<br>k_{cat}/K_m = 1,367 $S^{-1}M^{-1}$	K_i = 112.3 ± 8.57 nM	K_i = 37.18 ± 2.66 nM	IC_{50} = 175.2 ± 9.72 nM<br>K_i = 79.26 ± 4.13 nM	
L50F/ S144M		K_m = 88.44 ± 8.41 μM<br>V_{max} = 15.32 ± 0.68 nM/s<br>k_{cat} = 0.10 S^{-1}<br>k_{cat}/K_m = 1,155 $S^{-1}M^{-1}$	IC_{50} = 127.6 ± 19.6 nM	IC_{50} = 52.3 ± 7.7 nM	IC_{50} = 147.4 ± 21.9 nM<br>K_i = 33.84 ± 2.30 nM	

S144F	15	K_m = 45.11 ± 3.45 μM<br>V_{max} = 21.22 ± 0.61 nM/s<br>k_{cat} = 0.0849 S^{-1}<br>k_{cat}/K_m = 1,882 $S^{-1}M^{-1}$	K_i = 86.60 ± 8.39 nM	K_i = 26.37 ± 3.81 nM	IC_{50} = 133.3 ± 5.61 nM<br>K_i = 47.23 ± 2.24 nM	
S144P	12	K_m = 2.78 ± 0.58 μM<br>V_{max} = 0.58 ± 0.024 nM/s<br>k_{cat} = 0.000058 S^{-1}<br>k_{cat}/K_m = 21 $S^{-1}M^{-1}$			IC_{50} > 10 μM<br>K_i > 10 μM	
S144A	9	K_m = 25.66 ± 1.76 μM<br>V_{max} = 38.95 ± 1.12 nM/s<br>k_{cat} = 0.1558 S^{-1}<br>k_{cat}/K_m = 6,072 $S^{-1}M^{-1}$	IC_{50} = 139.80 ± 9.29 nM<br>K_i = 65.01 ± 4.76 nM	IC_{50} = 39.43 ± 1.14 nM<br>K_i = 12.61 ± 1.18 nM	IC_{50} = 171.1 ± 5.33 nM<br>K_i = 36.43 ± 4.11 nM	Apo: 8D4L<br>GC-376: 8D4M
S144W	7	K_m = 56.59 ± 3.01 μM<br>V_{max} = 15.55 ± 0.39 nM/s<br>k_{cat} = 0.0311 S^{-1}<br>k_{cat}/K_m = 550 $S^{-1}M^{-1}$			IC_{50} = 289.60 ± 14.78 nM<br>K_i = 143.80 ± 9.79 nM	
S144E	6	K_m = 52.69 ± 3.94 μM<br>V_{max} = 23.31 ± 0.69 nM/s<br>k_{cat} = 0.0233 S^{-1}<br>k_{cat}/K_m = 442 $S^{-1}M^{-1}$			IC_{50} = 423.40 ± 29.31 nM<br>K_i = 153.20 ± 9.74 nM	
S144V	4	K_m = 54.43 ± 3.58 μM<br>V_{max} = 17.12 ± 0.45 nM/s<br>k_{cat} = 0.0086 S^{-1}<br>k_{cat}/K_m = 157 $S^{-1}M^{-1}$			IC_{50} = 1,442 ± 127.2 nM<br>K_i = 858.9 ± 73.57 nM	
S144H	5	K_m = 60.72 ± 4.26 μM<br>V_{max} = 16.45 ± 0.48 nM/s<br>k_{cat} = 0.0329 S^{-1}<br>k_{cat}/K_m = 542 $S^{-1}M^{-1}$			IC_{50} = 650.5 ± 45.5 nM<br>K_i = 402.2 ± 19.92 nM	
S144Q	3	K_m = 64.17 ± 4.49 μM<br>V_{max} = 22.19 ± 0.65 nM/s<br>k_{cat} = 0.0222 S^{-1}<br>k_{cat}/K_m = 346 $S^{-1}M^{-1}$			IC_{50} = 831.2 ± 48.6 nM<br>K_i = 576.8 ± 54.29 nM	
S144R	2	K_m = 62.54 ± 5.09 μM<br>V_{max} = 11.57 ± 0.39 nM/s<br>k_{cat} = 0.0014 S^{-1}<br>k_{cat}/K_m = 23 $S^{-1}M^{-1}$			IC_{50} = 6,134 ± 704.8 nM<br>K_i = 2,190 ± 204.9 nM	
S144G	2	K_m = 53.52 ± 4.72 μM<br>V_{max} = 27.78 ± 0.97 nM/s<br>k_{cat} = 0.2222 S^{-1}<br>k_{cat}/K_m = 4,152 $S^{-1}M^{-1}$	K_i = 77.16 ± 7.56 nM	K_i = 16.52 ± 1.58 nM	IC_{50} = 96.55 ± 1.93 nM<br>K_i = 27.98 ± 2.76 nM	
S144Y	2	K_m = 55.15 ± 4.39 μM<br>V_{max} = 19.44 ± 0.62 nM/s<br>k_{cat} = 0.0778 S^{-1}<br>k_{cat}/K_m = 1,410 $S^{-1}M^{-1}$	K_i = 60.87 ± 4.93 nM	K_i = 18.08 ± 1.79 nM	IC_{50} = 61.49 ± 3.18 nM<br>K_i = 34.09 ± 3.67 nM	
S144K	2	K_m = 3.88 ± 0.31 μM<br>V_{max} = 0.80 ± 0.041 nM/s<br>k_{cat} = 0.00008 S^{-1}<br>k_{cat}/K_m = 21 $S^{-1}M^{-1}$			IC_{50} > 10 μM<br>K_i > 10 μM	
S144D	1	K_m = 27.60 ± 2.62 μM<br>V_{max} = 5.91 ± 0.18 nM/s<br>k_{cat} = 0.0035 S^{-1}<br>k_{cat}/K_m = 128 $S^{-1}M^{-1}$			IC_{50} = 8,532 ± 99.83 nM	
S144 deletion	84	K_m = 32.86 ± 1.76 μM<br>V_{max} = 10.74 ± 0.013 nM/s<br>k_{cat} = 0.0011 S^{-1}<br>k_{cat}/K_m = 33 $S^{-1}M^{-1}$				

H163W	4,673	Enzymatically inactive				
H164N	4,682	K_m = 30.72 ± 2.17 µM<br>V_{max} = 19.88 ± 0.69 nM/s<br>k_{cat} = 0.0795 S^{-1}<br>k_{cat}/K_m = 2,588 $S^{-1}M^{-1}$	IC_{50} = 126.3 ± 6.53 nM<br>K_i = 37.95 ± 2.58 nM	IC_{50} = 16.39 ± 0.92 nM<br>K_i = 3.45 ± 0.45 nM	IC_{50} = 32.85 ± 1.92 nM<br>K_i = 3.58 ± 0.43 nM	Apo: 8DFN<br>GC-376: 8DD1
H164 deletion		K_m = 36.98 ± 4.14 µM<br>V_{max} = 11.46 ± 0.17 nM/s<br>k_{cat} = 0.0011 S^{-1}<br>k_{cat}/K_m = 31 $S^{-1}M^{-1}$				
M165Y	4,678	K_m = 38.42 ± 2.32 µM<br>V_{max} = 20.26 ± 0.88 nM/s<br>k_{cat} = 0.0101 S^{-1}<br>k_{cat}/K_m = 264 $S^{-1}M^{-1}$	IC_{50} = 423.0 ± 22.3 nM<br>K_i = 164.6 ± 14.69 nM	IC_{50} = 286.2 ± 15.99 nM<br>K_i = 89.88 ± 8.11 nM	IC_{50} = 10,462 ± 497.7 nM<br>K_i = 7,216 ± 330.1 nM	Nirmatrelvir<br>8DCZ
M165L	280	K_m = 20.60 ± 4.24 µM<br>V_{max} = 17.54 ± 1.10 nM/s<br>k_{cat} = 0.2806 S^{-1}<br>k_{cat}/K_m = 13,623 $S^{-1}M^{-1}$			IC_{50} = 22.62 ± 0.77 nM<br>K_i =1.04 ± 0.079 nM	
M165I	101	K_m = 29.45 ± 4.87 µM<br>V_{max} = 14.90 ± 0.82 nM/s<br>k_{cat} = 0.2384 S^{-1}<br>k_{cat}/K_m = 8,095 $S^{-1}M^{-1}$			IC_{50} = 27.38 ± 2.09 nM<br>K_i =2.02 ± 0.16 nM	
M165V	16	K_m = 36.18 ± 2.61 µM<br>V_{max} = 33.75 ± 1.44 nM/s<br>k_{cat} = 0.3375 S^{-1}<br>k_{cat}/K_m = 9,328 $S^{-1}M^{-1}$			IC_{50} = 23.96 ± 0.72 nM	
M165W	11	K_m = 9.41 ± 0.92 µM<br>V_{max} = 1.34 ± 0.066 nM/s<br>k_{cat} = 0.0001 S^{-1}<br>k_{cat}/K_m = 12 $S^{-1}M^{-1}$			IC_{50} > 10 µM	
M165K	8	K_m = 36.41 ± 2.31 µM<br>V_{max} = 10.09 ± 0.33 nM/s<br>k_{cat} = 0.0012 S^{-1}<br>k_{cat}/K_m = 33 $S^{-1}M^{-1}$			IC_{50} >10 µM	
M165T	7	K_m = 55.37 ± 3.49 µM<br>V_{max} = 29.33 ± 0.74 nM/s<br>k_{cat} = 0.0733 S^{-1}<br>k_{cat}/K_m = 1,324 $S^{-1}M^{-1}$	IC_{50} = 239.6 ± 9.64 nM<br>K_i = 138.0 ± 7.86 nM	IC_{50} = 109.2 ± 4.92 nM<br>K_i = 33.58 ± 1.78 nM	IC_{50} = 94.68 ± 5.94 nM<br>K_i = 52.68 ± 4.68 nM	
M165R	5	K_m = 7.83 ± 0.76 µM<br>V_{max} = 2.32 ± 0.11 nM/s<br>k_{cat} = 0.0003 S^{-1}<br>k_{cat}/K_m = 33 $S^{-1}M^{-1}$			IC_{50} > 10 µM	
M165A	4	K_m = 50.82 ± 3.76 µM<br>V_{max} = 36.68 ± 1.99 nM/s<br>k_{cat} = 0.3668 S^{-1}<br>k_{cat}/K_m = 7,218 $S^{-1}M^{-1}$			IC_{50} = 23.28 ± 1.88 nM	
M165G	3	K_m = 56.00 ± 4.91 µM<br>V_{max} = 24.53 ± 0.86 nM/s<br>k_{cat} = 0.0245 S^{-1}<br>k_{cat}/K_m = 438 $S^{-1}M^{-1}$			IC_{50} = 243.5 ± 16.40 nM	
M165F	3	K_m = 74.74 ± 3.47 µM<br>V_{max} = 11.72 ± 0.24 nM/s<br>k_{cat} = 0.0059 S^{-1}<br>k_{cat}/K_m = 78 $S^{-1}M^{-1}$			IC_{50} = 1,336 ± 49.13 nM	

M165H	2	K_m = 14.71 ± 1.92 μM<br>V_{max} = 3.49 ± 0.13 nM/s<br>k_{cat} = 0.0003 S^{-1}<br>k_{cat}/K_m = 20 $S^{-1}M^{-1}$			IC_{50} > 10 μM	
M165P	2	K_m = 0.11 ± 0.024 μM<br>V_{max} = 1.02 ± 0.10 nM/s<br>k_{cat} = 0.0001 S^{-1}<br>k_{cat}/K_m = 773 $S^{-1}M^{-1}$			IC_{50} > 10 μM	
M165C	1	K_m = 46.24 ± 2.54 μM<br>V_{max} = 33.39 ± 0.96 nM/s<br>k_{cat} = 0.3339 S^{-1}<br>k_{cat}/K_m = 7,221 $S^{-1}M^{-1}$			IC_{50} = 24.84 ± 1.77 nM	
M165D	1	K_m = 68.73 ± 4.65 μM<br>V_{max} = 16.03 ± 0.46 nM/s<br>k_{cat} = 0.0178 S^{-1}<br>k_{cat}/K_m = 258 $S^{-1}M^{-1}$			IC_{50} = 147.6 ± 10.59 nM	
M165 deletion	67	K_m = 35.12 ± 4.13 μM<br>V_{max} = 2.30 ± 0.095 nM/s<br>k_{cat} = 0.0002 S^{-1}<br>k_{cat}/K_m = 7 $S^{-1}M^{-1}$				
E166Q	4,682	K_m = 36.04 ± 3.16 μM<br>V_{max} = 50.18 ± 1.55 nM/s<br>k_{cat} = 0.4014 S^{-1}<br>k_{cat}/K_m = 11,139 $S^{-1}M^{-1}$	IC_{50} = 52.31 ± 2.06 nM<br>K_i = 47.10 ± 3.49 nM	IC_{50} = 14.88 ± 0.47 nM<br>K_i = 4.45 ± 0.39 nM	IC_{50} = 27.87 ± 2.11 nM<br>K_i = 8.37 ± 0.74 nM	Apo: 8D4N
E166H	235	K_m = 55.96 ± 5.14 μM<br>V_{max} = 17.55 ± 0.65 nM/s<br>k_{cat} = 0.0351 S^{-1}<br>k_{cat}/K_m = 627 $S^{-1}M^{-1}$			IC_{50} = 9,598 ± 1,675 nM	
E166G	16	K_m = 32.90 ± 3.16 μM<br>V_{max} = 24.39 ± 0.98 nM/s<br>k_{cat} = 0.04878 S^{-1}<br>k_{cat}/K_m = 1,483 $S^{-1}M^{-1}$	K_i = 85.00 ± 8.16 nM	K_i = 13.54 ± 1.69 nM	IC_{50} = 92.15 ± 6.49 nM<br>K_i = 30.89 ± 2.55 nM	
E166K	10	K_m = 50.12 ± 4.68 μM<br>V_{max} = 25.93 ± 1.17 nM/s<br>k_{cat} = 0.02593 S^{-1}<br>k_{cat}/K_m = 517 $S^{-1}M^{-1}$			IC_{50} = 1,994 ± 175.1 nM	
E166V	8	K_m = 50.25 ± 2.81 μM<br>V_{max} = 25.01 ± 0.54 nM/s<br>k_{cat} = 0.0572 S^{-1}<br>k_{cat}/K_m = 1,138 $S^{-1}M^{-1}$	K_i = 584.4 ± 40.27 nM	K_i = 666.5 ± 73.39 nM	IC_{50} > 10,000 nM<br>K_i = 10,384 ± 673.3 nM	
E166A	5	K_m = 47.05 ± 2.49 μM<br>V_{max} = 17.16 ± 0.18 nM/s<br>k_{cat} = 0.0686 S^{-1}<br>k_{cat}/K_m = 1,459 $S^{-1}M^{-1}$	K_i = 139.6 ± 14.22 nM	K_i = 22.64 ± 2.49 nM	IC_{50} = 153.0 ± 10.28 nM<br>K_i = 89.29 ± 6.47 nM	
E166L	3	K_m = 18.73 ± 0.99 μM<br>V_{max} = 2.16 ± 0.034 nM/s<br>k_{cat} = 0.000432 S^{-1}<br>k_{cat}/K_m = 23 $S^{-1}M^{-1}$			IC_{50} = 214.2 ± 18.57 nM	
E166Y	2	K_m = 27.92 ± 1.67 μM<br>V_{max} = 11.00 ± 0.43 nM/s<br>k_{cat} = 0.011 S^{-1}<br>k_{cat}/K_m = 394 $S^{-1}M^{-1}$			IC_{50} = 3,711 ± 283.1 nM	
E166I	1	K_m = 37.82 ± 3.77 μM<br>V_{max} = 2.00 ± 0.14 nM/s<br>k_{cat} = 0.0002 S^{-1}<br>k_{cat}/K_m = 5 $S^{-1}M^{-1}$			IC_{50} > 10,000 nM	

E166 deletion	67	K_m = 33.97 ± 2.46 μM<br>V_{max} = 1.92 ± 0.048 nM/s<br>k_{cat} = 0.0002 S^{-1}<br>k_{cat}/K_m = 6 $S^{-1}M^{-1}$				
H172T	99	K_m = 51.84 ± 3.01 μM<br>V_{max} = 6.29 ± 0.14 nM/s<br>k_{cat} = 0.0006 S^{-1}<br>k_{cat}/K_m = 12 $S^{-1}M^{-1}$	IC_{50} > 10,000 nM<br>K_i = 6,842 ± 657.1 nM	IC_{50} = 7,888 ± 420 nM<br>K_i = 1,293 ± 95.15 nM	IC_{50} > 10,000 nM<br>K_i = 12,672 ± 545.4 nM	
H172Y	21	K_m = 39.20 ± 2.83 μM<br>V_{max} = 15.26 ± 0.39 nM/s<br>k_{cat} = 0.0305 S^{-1}<br>k_{cat}/K_m = 790 $S^{-1}M^{-1}$	IC_{50} = 449.2 ± 27.81 nM<br>K_i = 432.5 ± 17.40 nM	IC_{50} = 126.4 ± 5.64 nM<br>K_i = 57.06 ± 5.79 nM	IC_{50} = 279.3 ± 23.09 nM<br>K_i = 275.1 ± 10.83 nM	Apo: 8D4J<br>GC-376: 8D4K
H172E	16	K_m = 13.43 ± 0.88 μM<br>V_{max} = 3.23 ± 0.12 nM/s<br>k_{cat} = 0.000323 S^{-1}<br>k_{cat}/K_m = 24 $S^{-1}M^{-1}$			IC_{50} > 10,000 nM	
H172Q	12	K_m = 49.61 ± 4.41 μM<br>V_{max} = 21.53 ± 0.74 nM/s<br>k_{cat} = 0.1722 S^{-1}<br>k_{cat}/K_m = 3,472 $S^{-1}M^{-1}$	K_i = 214.4 ± 11.36 nM	K_i = 42.66 ± 3.56 nM	IC_{50} = 152.1 ± 8.01 nM<br>K_i = 70.54 ± 3.90 nM	
H172D	10	K_m = 53.01 ± 4.79 μM<br>V_{max} = 13.87 ± 0.50 nM/s<br>k_{cat} = 0.0277 S^{-1}<br>k_{cat}/K_m = 523 $S^{-1}M^{-1}$			IC_{50} = 266.1 ± 24.8 nM<br>K_i = 170.6 ± 11.30 nM	
H172L	8	K_m = 54.89 ± 2.08 μM<br>V_{max} = 5.99 ± 0.11 nM/s<br>k_{cat} = 0.0012 S^{-1}<br>k_{cat}/K_m = 22 $S^{-1}M^{-1}$			IC_{50} = 3,380 ± 295.48 nM<br>K_i = 3,135 ± 227.7 nM	
H172M	7	K_m = 60.05 ± 3.65 μM<br>V_{max} = 19.62 ± 0.49 nM/s<br>k_{cat} = 0.0196 S^{-1}<br>k_{cat}/K_m = 327 $S^{-1}M^{-1}$			IC_{50} = 699.0 ± 27.0 nM<br>K_i = 290.6 ± 18.98 nM	
H172A	7	K_m = 56.15 ± 3.40 μM<br>V_{max} = 27.33 ± 0.67 nM/s<br>k_{cat} = 0.0547 S^{-1}<br>k_{cat}/K_m = 973 $S^{-1}M^{-1}$			IC_{50} = 374.7 ± 30.5 nM<br>K_i = 213.8 ± 13.47 nM	
H172F	5	K_m = 66.53 ± 2.68 μM<br>V_{max} = 29.64 ± 0.51 nM/s<br>k_{cat} = 0.0741 S^{-1}<br>k_{cat}/K_m = 1,114 $S^{-1}M^{-1}$	K_i = 188.5 ± 12.41 nM	K_i = 36.00 ± 2.59 nM	IC_{50} = 212.2 ± 9.69 nM<br>K_i = 46.60 ± 5.59 nM	
H172I	5	K_m = 61.31 ± 4.27 μM<br>V_{max} = 58.34 ± 1.68 nM/s<br>k_{cat} = 0.00583 S^{-1}<br>k_{cat}/K_m = 95 $S^{-1}M^{-1}$			IC_{50} > 10 μM<br>K_i > 10 μM	
H172V	5	K_m = 37.81 ± 1.79 μM<br>V_{max} = 7.14 ± 0.12 nM/s<br>k_{cat} = 0.0014 S^{-1}<br>k_{cat}/K_m = 38 $S^{-1}M^{-1}$			IC_{50} > 10 μM	
H172N	5	K_m = 62.46 ± 2.12 μM<br>V_{max} = 15.33 ± 0.22 nM/s<br>k_{cat} = 0.0051 S^{-1}<br>k_{cat}/K_m = 82 $S^{-1}M^{-1}$			IC_{50} = 1,827 ± 337.24 nM<br>K_i = 1,613 ± 60.97 nM	
H172S	4	K_m = 65.48 ± 2.33 μM<br>V_{max} = 15.5 ± 0.23 nM/s<br>k_{cat} = 0.0052 S^{-1}<br>k_{cat}/K_m = 79 $S^{-1}M^{-1}$			IC_{50} = 5,650 ± 180.8 nM<br>K_i = 2,204 ± 142.8 nM	

H172K	3	K_m = 55.53 ± 2.57 μM<br>V_{max} = 16.12 ± 0.29 nM/s<br>k_{cat} = 0.0107 S^{-1}<br>k_{cat}/K_m = 194 $S^{-1}M^{-1}$			IC_{50} = 1,030 ± 50.53 nM	
H172R	1	K_m = 1.09 ± 0.15 μM<br>V_{max} = 1.19 ± 0.037 nM/s<br>k_{cat} = 0.00036 S^{-1}<br>k_{cat}/K_m = 262 $S^{-1}M^{-1}$			IC_{50} >10 μM	
H172G	1	K_m = 74.20 ± 2.31 μM<br>V_{max} = 9.95 ± 0.14 nM/s<br>k_{cat} = 0.0119 S^{-1}<br>k_{cat}/K_m = 161 $S^{-1}M^{-1}$			IC_{50} = 810.2 ± 19.30 nM	
H172C	1	K_m = 66.79 ± 3.00 μM<br>V_{max} = 12.59 ± 0.24 nM/s<br>k_{cat} = 0.0094 S^{-1}<br>k_{cat}/K_m = 141 $S^{-1}M^{-1}$			IC_{50} = 3,056 ± 158.3 nM	
H172 deletion	59	K_m = 39.14 ± 6.23 μM<br>V_{max} = 6.81 ± 0.39 nM/s<br>k_{cat} = 0.0007 S^{-1}<br>k_{cat}/K_m = 17 $S^{-1}M^{-1}$				
Q189K	168	K_m = 41.45 ± 2.66 μM<br>V_{max} = 39.78 ± 1.01 nM/s<br>k_{cat} = 0.16 S^{-1}<br>k_{cat}/K_m = 3,800 $S^{-1}M^{-1}$	IC_{50} = 73.65 ± 4.09 nM<br>K_i = 64.09 ± 3.45 nM	IC_{50} = 37.23 ± 0.83 nM<br>K_i = 14.35 ± 1.65 nM	IC_{50} = 39.31 ± 1.92 nM<br>K_i = 14.60 ± 1.53 nM	
Q189F	39	K_m = 26.02 ± 2.40 μM<br>V_{max} = 19.49 ± 1.16 nM/s<br>k_{cat} = 0.3118 S^{-1}<br>k_{cat}/K_m = 11,985 $S^{-1}M^{-1}$			IC_{50} = 34.02 ± 1.47 nM	
Q189R	27	K_m = 42.63 ± 2.72 μM<br>V_{max} = 21.84 ± 0.52 nM/s<br>k_{cat} = 0.1092 S^{-1}<br>k_{cat}/K_m = 2,562 $S^{-1}M^{-1}$			IC_{50} = 37.82 ± 1.16 nM	
Q189H	20	K_m = 42.83 ± 3.24 μM<br>V_{max} = 18.05 ± 0.51 nM/s<br>k_{cat} = 0.1444 S^{-1}<br>k_{cat}/K_m = 3,371 $S^{-1}M^{-1}$			IC_{50} = 32.71 ± 1.04 nM	
Q189L	19	K_m = 47.00 ± 3.83 μM<br>V_{max} = 15.42 ± 0.48 nM/s<br>k_{cat} = 0.0617 S^{-1}<br>k_{cat}/K_m = 1,312 $S^{-1}M^{-1}$			IC_{50} = 39.25 ± 1.47 nM	
Q189P	18	K_m = 29.57 ± 2.25 μM<br>V_{max} = 19.07 ± 0.97 nM/s<br>k_{cat} = 0.3051 S^{-1}<br>k_{cat}/K_m = 10,319 $S^{-1}M^{-1}$			IC_{50} = 14.08 ± 0.70 nM	
Q189S	8	K_m = 27.97 ± 3.26 μM<br>V_{max} = 17.28 ± 0.66 nM/s<br>k_{cat} = 0.1728 S^{-1}<br>k_{cat}/K_m = 6,178 $S^{-1}M^{-1}$			IC_{50} = 25.34 ± 1.72 nM	
Q189E	8	K_m = 23.03 ± 1.71 μM<br>V_{max} = 29.75 ± 1.91 nM/s<br>k_{cat} = 0.4760 S^{-1}<br>k_{cat}/K_m = 20,669 $S^{-1}M^{-1}$	IC_{50} = 80.19 ± 6.31 nM<br>K_i = 36.86 ± 2.97 nM	IC_{50} = 42.34 ± 3.07 nM<br>K_i = 1.34 ± 0.14 nM	IC_{50} = 49.71 ± 3.19 nM<br>K_i = 4.51 ± 0.29 nM	
Q189 deletion	1093	K_m = 59.36 ± 5.73 μM<br>V_{max} = 11.67 ± 0.14 nM/s<br>k_{cat} = 0.0039 S^{-1}<br>k_{cat}/K_m = 66 $S^{-1}M^{-1}$				

Q192T	187	K_m = 32.94 ± 2.96 μM<br>V_{max} = 19.94 ± 0.62 nM/s<br>k_{cat} = 0.0399 S^{-1}<br>k_{cat}/K_m = 1,200 $S^{-1}M^{-1}$	IC_{50} = 237.2 ± 17.12 nM<br>K_i = 119.7 ± 6.57 nM	IC_{50} = 185.5 ± 13.90 nM<br>K_i = 71.40 ± 4.26 nM	IC_{50} = 102.6 ± 5.52 nM<br>K_i = 45.69 ± 4.43 nM	GC-376: 8DGB
Q192K	63	K_m = 38.29 ± 2.05 μM<br>V_{max} = 12.21 ± 0.23 nM/s<br>k_{cat} = 0.0122 S^{-1}<br>k_{cat}/K_m = 319 $S^{-1}M^{-1}$	IC_{50} = 303.6 ± 27.42 nM<br>K_i = 430.3 ± 22.35 nM	IC_{50} = 324.0 ± 18.93 nM<br>K_i = 299.5 ± 23.79 nM	IC_{50} = 219.1 ± 9.62 nM<br>K_i = 88.90 ± 4.88 nM	
Q192S	29	K_m = 46.24 ± 2.09 μM<br>V_{max} = 28.69 ± 0.49 nM/s<br>k_{cat} = 0.0574 S^{-1}<br>k_{cat}/K_m = 1,241 $S^{-1}M^{-1}$	IC_{50} = 319.0 ± 12.02 nM<br>K_i = 227.9 ± 17.06 nM	IC_{50} = 183.9 ± 8.16 nM<br>K_i = 82.32 ± 4.23 nM	IC_{50} = 217.3 ± 10.59 nM<br>K_i = 75.56 ± 8.45 nM	
Q192L	12	K_m = 62.36 ± 3.76 μM<br>V_{max} = 31.72 ± 0.79 nM/s<br>k_{cat} = 0.1586 S^{-1}<br>k_{cat}/K_m = 2,543 $S^{-1}M^{-1}$	K_i = 165.3 ± 17.86 nM	K_i = 25.39 ± 1.81 nM	IC_{50} = 121.9 ± 5.51 nM<br>K_i = 70.99 ± 7.02 nM	
Q192R	12	K_m = 59.39 ± 4.71 μM<br>V_{max} = 17.70 ± 0.57 nM/s<br>k_{cat} = 0.0283 S^{-1}<br>k_{cat}/K_m = 477 $S^{-1}M^{-1}$			IC_{50} = 229.6 ± 11.54 nM	
Q192N	9	K_m = 54.93 ± 3.37 μM<br>V_{max} = 9.19 ± 0.22 nM/s<br>k_{cat} = 0.0408 S^{-1}<br>k_{cat}/K_m = 744 $S^{-1}M^{-1}$			IC_{50} = 73.69 ± 4.96 nM	
Q192A	9	K_m = 59.42 ± 3.02 μM<br>V_{max} = 35.25 ± 0.0.73 nM/s<br>k_{cat} = 0.1058 S^{-1}<br>k_{cat}/K_m = 1,780 $S^{-1}M^{-1}$	K_i = 206.8 ± 14.64 nM	K_i = 62.84 ± 5.63 nM	IC_{50} = 140.7 ± 3.93 nM<br>K_i = 58.10 ± 5.67 nM	
Q192I	8	K_m = 52.72 ± 4.87 μM<br>V_{max} = 21.47 ± 0.78 nM/s<br>k_{cat} = 0.1031 S^{-1}<br>k_{cat}/K_m = 1,955 $S^{-1}M^{-1}$	K_i = 179.8 ± 10.86 nM	K_i = 41.16 ± 5.37 nM	IC_{50} = 109.9 ± 2.45 nM<br>K_i = 43.53 ± 3.13 nM	
Q192P	8	K_m = 55.88 ± 3.74 μM<br>V_{max} = 20.34 ± 0.55 nM/s<br>k_{cat} = 0.0814 S^{-1}<br>k_{cat}/K_m = 1,456 $S^{-1}M^{-1}$	K_i = 217.1 ± 19.24 nM	K_i = 80.95 ± 8.68 nM	IC_{50} = 135.1 ± 7.17 nM<br>K_i = 76.19 ± 5.66 nM	
Q192G	7	K_m = 68.11 ± 2.55 μM<br>V_{max} = 20.69 ± 0.33 nM/s<br>k_{cat} = 0.0414 S^{-1}<br>k_{cat}/K_m = 608 $S^{-1}M^{-1}$	K_i = 277.6 ± 19.21 nM	K_i = 78.78 ± 7.01 nM	IC_{50} = 319.1 ± 12.73 nM<br>K_i = 106.3 ± 5.84 nM	
Q192H	7	K_m = 60.51 ± 3.86 μM<br>V_{max} = 27.12 ± 0.71 nM/s<br>k_{cat} = 0.0814 S^{-1}<br>k_{cat}/K_m = 1,345 $S^{-1}M^{-1}$	K_i = 237.2 ± 21.98 nM	K_i = 61.19 ± 3.93 nM	IC_{50} = 169.4 ± 7.27 nM<br>K_i = 80.69 ± 7.33 nM	
Q192Y	7	K_m = 9.18 ± 0.62 μM<br>V_{max} = 2.64 ± 0.12 nM/s<br>k_{cat} = 0.0004 S^{-1}<br>k_{cat}/K_m = 43 $S^{-1}M^{-1}$			IC_{50} >10 μM	
Q192V	6	K_m = 59.77 ± 3.01 μM<br>V_{max} = 18.36 ± 0.38 nM/s<br>k_{cat} = 0.0734 S^{-1}<br>k_{cat}/K_m = 1,229 $S^{-1}M^{-1}$	IC_{50} = 136.1 ± 5.66 nM<br>K_i = 122.0 ± 9.62 nM	IC_{50} = 82.17 ± 2.54 nM<br>K_i = 78.46 ± 6.47 nM	IC_{50} = 95.58 ± 4.23 nM<br>K_i = 30.87 ± 1.12 nM	
Q192W	5	K_m = 50.15 ± 4.32 μM<br>V_{max} = 24.00 ± 0.80 nM/s<br>k_{cat} = 0.0691 S^{-1}<br>k_{cat}/K_m = 1,378 $S^{-1}M^{-1}$	K_i = 149.0 ± 12.78 nM	K_i = 32.88 ± 2.89 nM	IC_{50} = 65.94 ± 2.54 nM<br>K_i = 43.65 ± 2.32 nM	

Q192E	5	K_m = 60.37 ± 3.88 μM<br>V_{max} = 32.98 ± 0.87 nM/s<br>k_{cat} = 0.0660 S^{-1}<br>k_{cat}/K_m = 1,093 $S^{-1}M^{-1}$	K_i = 296.1 ± 21.17 nM	K_i = 39.21 ± 2.92 nM	IC_{50} = 193.1 ± 9.27 nM<br>K_i = 114.4 ± 10.82 nM	
Q192C	3	K_m = 55.53 ± 3.46 μM<br>V_{max} = 21.80 ± 0.54 nM/s<br>k_{cat} = 0.0872 S^{-1}<br>k_{cat}/K_m = 1,570 $S^{-1}M^{-1}$	K_i = 147.7 ± 16.25 nM	K_i = 27.98 ± 1.58 nM	IC_{50} = 96.82 ± 2.40 nM<br>K_i = 54.12 ± 2.79 nM	
Q192D	3	K_m = 68.35 ± 5.76 μM<br>V_{max} = 18.58 ± 0.67 nM/s<br>k_{cat} = 0.0372 S^{-1}<br>k_{cat}/K_m = 544 $S^{-1}M^{-1}$			IC_{50} = 271.5 ± 17.63 nM	
Q192F	2	K_m = 54.41 ± 4.34 μM<br>V_{max} = 42.44 ± 1.35 nM/s<br>k_{cat} = 0.1698 S^{-1}<br>k_{cat}/K_m = 3,120 $S^{-1}M^{-1}$	K_i = 137.5 ± 11.09 nM	K_i = 21.41 ± 2.28 nM	IC_{50} = 92.68 ± 6.53 nM<br>K_i = 85.50 ± 12.51 nM	
Q192 deletion	1070	K_m = 34.20 ± 4.24 μM<br>V_{max} = 3.00 ± 0.13 nM/s<br>k_{cat} = 0.0008 S^{-1}<br>k_{cat}/K_m = 22 $S^{-1}M^{-1}$				
Q189E/ H172Y	0	K_m = 61.34 ± 4.83 μM<br>V_{max} = 24.76 ± 0.80 nM/s<br>k_{cat} = 0.0619 S^{-1}<br>k_{cat}/K_m = 1,009 $S^{-1}M^{-1}$	IC_{50} = 652.7 ± 30.98 nM<br>K_i = 618.2 ± 36.42 nM	IC_{50} = 169.9 ± 11.54 nM<br>K_i = 83.84 ± 4.61 nM	IC_{50} = 603.7 ± 17.42 nM<br>K_i = 528.5 ± 47.25 nM	

[a]The occurrence of mutation was analyzed using the GISAID CoVsurver as of September 14th, 2022 (latest update on July 7, 2022). k_{cat}, V_{max}, K_m, k_{cat}/K_m, IC_{50} and K_i values are the average of two repeats. Enzymatically inactive mutants are colored in yellow. M^{pro} mutants that have comparable enzymatic activity as the WT (k_{cat}/K_m <10-fold change) and are resistant to nirmatrelvir (K_i > 10-fold increase) are colored in red.

Table S2. Thermal shift assay results of nirmatrelvir with M^{pro} mutants.

nirmatrelvir (μM)	ΔTm (°C)										
	WT	S144M	S144F	S144A	S144G	S144Y	M165T	E166A	E166G	H172Q	H172F
200	19.95	12.91	16.55	15.96	15.16	17.05	15.40	12.34	17.34	12.21	12.90
60	19.07	10.45	13.67	14.39	12.00	14.10	12.60	10.23	14.71	9.26	10.94
20	17.97	6.81	10.94	12.94	8.35	11.23	9.94	6.05	11.25	5.33	8.41
6	11.65	1.26	7.71	8.71	1.82	8.49	5.95	2.90	7.34	1.19	5.47
2	9.40	0.56	1.68	5.65	0.42	3.58	1.40	1.41	2.65	0.28	1.47
0.6	2.33	0.28	0.56	1.79	0.14	0.28	0.28	1.20	1.12	-0.07	0.49
0.2	0.68	0.14	0.21	0.31	-0.14	-0.14	-0.07	0.00	0.06	-0.14	0.14

nirmatrelvir (μM)	ΔTm (°C)										
	Q192T	Q192S	Q192L	Q192A	Q192I	Q192P	Q192H	Q192V	Q192W	Q192C	Q192F
200	15.36	15.01	16.49	13.53	17.10	16.20	16.17	16.95	15.49	15.74	14.94
60	12.28	11.99	12.89	12.13	13.67	13.75	12.81	14.43	13.17	11.80	13.43
20	9.12	8.42	9.13	8.74	10.94	10.45	8.89	11.35	9.89	8.12	10.00
6	4.98	3.65	5.55	5.50	5.54	5.75	1.82	7.22	6.46	4.77	6.41
2	2.03	1.75	2.23	2.28	1.33	1.40	0.56	2.24	2.04	1.65	2.35
0.6	0.77	0.70	0.71	0.91	0.49	0.28	0.28	0.63	0.10	0.00	0.95
0.2	-0.07	0.28	0.07	0.44	0.14	0.14	0.07	0.14	0.03	0.02	0.39

Table S3. X-ray Data Collection and Refinement Statistics

Data Collection											
Structure (PDB ID)	8D4J	8D4K	8D4L	8D4M	8D4N	8DFN	8DD1	8DFE	8DD9	8DGB	8DCZ
Mutation	H172Y	H172Y	S144A	S144A	E166Q	H164N	H164N	S144L	S144L	Q192T	M165Y
Ligand	Apo	GC376	Apo	GC376	Apo	Apo	GC376	Apo	GC376	GC376	Nirmatrelvir
Space Group	$P2_1$	C2	$P2_1$	C2	$P2_1$	$P2_1$	C2	C2	C2	P1	$P2_1$
Cell Dimensions											
a, *b*, *c* (Å)	44.962	114.549	44.625	114.134	44.816	44.668	113.899	113.554	113.707	47.03	45.566
	53.474	53.111	53.707	53.653	53.687	53.738	53.55	54.075	53.153	53.241	53.786
	114.185	45.643	114.685	45.457	114.532	114.781	45.321	44.615	45.345	59.532	114.76
α, β, γ (°)	90	90	90	90	90	90	90	90	90	67	90
	101.14	102.67	101.3	101.97	101.79	100.81	101.9	100.73	102.22	79.57	100.53
	90	90	90	90	90	90	90	90	90	88.8	90
Resolution (Å)	50-1.78	50-1.89	50-1.70	50-1.81	50-2.70	50-2.04	50-2.04	50-1.89	50-2.04	50-2.87	50-2.38
No. Reflections	51114	21112	56762	24143	14631	33845	16735	20077	16416	10878	21947
R_{merge}(%)	6.3	8.4	5.5	7.4	17.2	8.7	7.8	7.1	9.7	7.9	9.2
I / $\acute{o}I$	24.2 (2.73)	20.35 (2.01)	28.72 (3.46)	21.77 (2.34)	9.13 (2.02)	16.75 (2.14)	22.18 (2.27)	26.7 (2.63)	13.59 (2.31)	9.03 (3.06)	12.33 (2.13)
Completeness (%)	99.5	98.6	96.8	98.1	99.1	98.6	97.0	94.0	96.9	93.5	98.6
Redundancy	3.5 (2.6)	4.1 (3.9)	3.4 (3.2)	3.9 (4.1)	3.7 (3.6)	5.2 (5.2)	4.9 (4.7)	6.2 (5.9)	3.7 (3.3)	2.2 (1.5)	4.0 (3.5)
**Refinement**											
Resolution (Å)	38.71-1.78	48.02-1.89	38.87-1.70	48.41-1.81	38.80-2.70	43.79-2.04	34.24-2.04	48.41-1.89	39.92-2.04	48.98-2.87	39.28-2.38
R_{work}/R_{free} (%)	16.6/20.9	16.6/22.4	17.1/21.3	17.2/22.9	20.6/25.8	19.1/25.1	18.5/24.8	20.8/26.8	16.5/22.8	22.7/27.2	19.2/25.1
**No. Heavy Atoms**											
Protein	4912	2443	4912	2431	4844	4703	2345	2421	2411	4723	4757
Ligand/Ion	6	29	0	29	0	0	29	0	29	58	70
Water	478	187	471	246	40	304	148	201	208	11	140
***B*-Factors (Å^2)**											
Protein	27.19	30.87	25.91	31.04	34.42	33.18	45.22	30.96	33.51	56.10	40.08
Ligand/Ion	36.91	30.63	0	32.29	0	0	40.87	0	25.19	59.20	31.48
Water	34.53	37.51	33.21	37.49	20.3	36.22	48.83	34.38	38.29	54.78	37.38
**Ramanchandran Plot**											
Favored Region (%)	98.5	98.0	98.5	98.7	98.7	98.3	98.3	98.7	98.7	97.4	97.7
Allowed Region (%)	1.5	2.0	1.5	1.3	1.0	1.3	1.3	1.3	1.3	2.6	2.0
Outlier Region (%)	0.0	0.0	0.0	0.0	0.3	0.3	0.3	0.0	0.0	0.0	0.3

*values in the parentheses represent highest resolution shells

Table S4. Protein concentrations used for the K_m/V_{max}, IC_{50} and K_i measurements.

M^{pro} mutants	M^{pro} concentration (μM)		
	K_m/V_{max}	IC_{50}	K_i
WT	0.1	0.1	0.005
H41M	10		
H41T	10		
H41Y	10		
M49I	0.05	0.1	0.025
M49T	0.0625	0.1	0.005
M49L	0.0625	0.1	0.003
M49V	0.125	0.125	
M49 deletion	0.1	0.1	0.05
T135I	0.1	0.1	0.05
T135 deletion	5		
N142S	0.1	0.1	
N142D	0.125	0.125	
N142L	0.0625	0.1	
N142I	0.0625	0.1	
N142H	0.125	0.125	
N142M	0.0625	0.1	
N142K	0.0625	0.1	
N142T	0.0625	0.1	
N142Y	0.0625	0.1	
N142 deletion	5		
S144L	2	2	0.5
S144T	0.75	0.75	0.25
S144M	0.4	0.4	0.125
L50F/S144M	0.15	0.15	0.05
S144F	0.25	0.25	0.125
S144P	10	10	5

S144A	0.25	0.25	0.04
S144W	0.5	0.5	0.25
S144E	1	1	0.2
S144V	2	2	1
S144H	0.5	0.5	0.25
S144Q	1	1	0.5
S144R	8	8	4
S144G	0.125	0.125	0.05
S144Y	0.25	0.25	0.125
S144K	10	10	2
S144D	2	2	
S144 deletion	10		
H163W	10		
H164N	0.25	0.25	0.1
H164 deletion	10		
M165Y	2	2	0.25
M165L	0.0625	0.1	0.003
M165I	0.0625	0.1	0.005
M165V	0.1	0.1	
M165W	12	12	
M165K	10	10	
M165T	0.4	0.4	0.15
M165R	12	12	
M165A	0.1	0.1	
M165G	1	1	
M165F	2	2	
M165H	12	12	
M165P	12	12	
M165C	0.1	0.1	

M165D	1.2	1.2	
M165 deletion	10		
E166Q	0.125	0.125	0.0625
E166H	0.5	0.5	
E166G	0.5	0.5	0.2
E166K	1	1	
E166V	0.4	0.4	0.2
E166A	0.25	0.25	0.125
E166L	5	5	
E166Y	1	1	
E166I	10	10	
E166 deletion	10		
H172T	10	10	2
H172Y	0.5	0.5	0.15
H172E	10	10	
H172Q	0.125	0.125	0.0625
H172D	0.5	0.5	0.25
H172L	5	5	2
H172M	1	1	0.4
H172A	0.5	0.5	0.25
H172F	0.4	0.4	0.125
H172I	10	10	5
H172V	5	5	
H172N	3	3	1
H172S	3	3	1
H172K	1.5	1.5	
H172R	5	5	
H172G	1	1	
H172C	2	2	

H172 deletion	10		
Q189K	0.25	0.25	0.05
Q189F	0.0625	0.1	
Q189R	0.2	0.2	
Q189H	0.125	0.125	
Q189L	0.25	0.25	
Q189P	0.0625	0.1	
Q189S	0.1	0.1	
Q189E	0.0625	0.1	0.01
Q189 deletion	3	3	
Q192T	0.5	0.5	0.2
Q192K	1	1	0.25
Q192S	0.5	0.5	0.25
Q192L	0.3	0.3	0.15
Q192R	1	1	
Q192N	0.3	0.3	
Q192A	0.5	0.5	0.175
Q192I	0.25	0.25	0.15
Q192P	0.3	0.3	0.15
Q192G	0.75	0.75	0.25
Q192H	0.5	0.5	0.175
Q192Y	10	10	
Q192V	0.25	0.25	0.125
Q192W	0.5	0.5	0.25
Q192E	0.5	0.5	0.2
Q192C	0.25	0.25	0.125
Q192D	0.5	0.5	
Q192F	0.25	0.25	0.125
Q192 deletion	4		

Q189E/H172Y	0.4	0.4	0.1
